# Overexpression of Acyl-ACP Thioesterases, *CpFatB4* and *CpFatB5*, Induce Distinct Gene Expression Reprogramming in Developing Seeds of *Brassica napus*

**DOI:** 10.3390/ijms20133334

**Published:** 2019-07-06

**Authors:** Jeong-Won Nam, Jinouk Yeon, Jiseong Jeong, Eunyoung Cho, Ho Bang Kim, Yoonkang Hur, Kyeong-Ryeol Lee, Hankuil Yi

**Affiliations:** 1 Department of Biological Sciences, Chungnam National University, Daejeon 34134, Korea; 2 Life Sciences Research Institute, Biomedic Co., Ltd., Bucheon 14548, Korea; 3 Department of Agricultural Biotechnology, National Agricultural Science, RDA, Jeonju 55365, Korea

**Keywords:** *Brassica napus*, lipid metabolism, acyl-ACP thioesterase, saturated fatty acid, RNA-Seq

## Abstract

We examined the substrate preference of *Cuphea paucipetala* acyl-ACP thioesterases, *CpFatB4* and *CpFatB5*, and gene expression changes associated with the modification of lipid composition in the seed, using *Brassica napus* transgenic plants overexpressing *CpFatB4* or *CpFatB5* under the control of a seed-specific promoter. *CpFatB4* seeds contained a higher level of total saturated fatty acid (FA) content, with 4.3 times increase in 16:0 palmitic acid, whereas *CpFatB5* seeds showed approximately 3% accumulation of 10:0 and 12:0 medium-chain FAs, and a small increase in other saturated FAs, resulting in higher levels of total saturated FAs. RNA-Seq analysis using entire developing pods at 8, 25, and 45 days after flowering (DAF) showed up-regulation of genes for β-ketoacyl-acyl carrier protein synthase I/II, stearoyl-ACP desaturase, oleate desaturase, and linoleate desaturase, which could increase unsaturated FAs and possibly compensate for the increase in 16:0 palmitic acid at 45 DAF in *CpFatB4* transgenic plants. In *CpFatB5* transgenic plants, many putative chloroplast- or mitochondria-encoded genes were identified as differentially expressed. Our results report comprehensive gene expression changes induced by alterations of seed FA composition and reveal potential targets for further genetic modifications.

## 1. Introduction

*Brassica napus* (rapeseed) is one of the most-produced oilseed crops in the world, second only to soybean, and is used for human consumption, biodiesel, and industrial raw materials [1]. The oil content in the seeds of currently cultivated *B. napus* is approximately 40% of the seed weight, and the fatty acid (FA) composition is up to 70% 18:1 oleic acid. The FA compositions of *B. napus* and other temperate oilseed crops are generally high in unsaturated FAs [2]. Unsaturated FAs are appreciated in the nutritional sector because of their beneficial health effects. However, polyunsaturated FAs are susceptible to oxidation and thus have a limited shelf-life. On the other hand, saturated FAs also have many valuable uses, especially in the industrial sector for the manufacture of soaps, cosmetics, and lubricants. Unlike many temperate oilseed crops, the tropical palm tree has a high content of saturated FAs in its oil. Palm oil is a widely used vegetable oil which comprises 44% 16:0 FAs and a total of 50% saturated FAs [3]. Although the oil palm is an efficient crop, producing up to ten times more oil per hectare than soybean, *B. napus*, or sunflower, palm trees grow only in the limited area of the tropical zone, and palm oil production leads to tropical deforestation [4]. Therefore, it would be beneficial to modify the composition of saturated FAs in *B. napus* growing in the temperate area. The *Agrobacterium*-mediated transformation method for *B. napus* is well established, and it allows the manipulation of seed oil composition [5].

*B. napus* is an allopolyploid plant with ample genetic, genomic, and transcriptomic information [6,7]. It contains 101,040 gene models and 1097 and 1132 lipid biosynthesis genes annotated in the A and C subgenomes, respectively [8]. In addition, various transcriptome analyses, including those for seed development and oil metabolism, have been conducted for *B. napus*. Troncoso-Ponce et al. [9] performed comparative profiling for *B. napus* and three other oilseeds using deep expressed sequence tags (ESTs), and Roh et al. [10] reported the gene expression profiling of *B. napus* embryos using microarray. Candidates for lipid-related genes in *B. napus* pods have been identified using RNA-Seq transcriptome analysis, and the expression of lipid biosynthesis genes in leaves and developing seeds has been compared [11,12]. In *Brassica napus*, the total lipid content in seeds accounted for 37% of the total dry weight of seeds, but only 6.1% of the total dry weight in leaves [12]. Simultaneous analyses of developing seed transcriptomes and proteomes at 2, 4, 6, and 8 weeks after pollination (WAP) revealed that FA biosynthesis and unsaturation are predominant biological processes from 2–4 and 4–6 WAP, respectively [13].

Many efforts have been made to increase the saturated FA content in *B. napus*. Early efforts included the genetic engineering of stearoyl-ACP desaturase (SAD), acyl-ACP thioesterases, β-ketoacyl-ACP synthases, and acyltransferases [14]. For example, overexpression of *Umbellularia californica* 12:0-ACP thioesterase (*UcFatB1*) generated transgenic *B. napus* seeds containing about 60% of 12:0 FA [15]. Coexpression of UcFatB1 and coconut (*Cocos nucifera*) lysophosphatidic acid acyltransferase with a preference for 12:0-CoA in *B. napus* further increased 12:0 FA accumulation up to 67% [16]. An increase of 16:0 and saturated FA content up to 31% and 46% was respectively achieved using a combination of SAD silencing and native fatty acyl-ACP thioesterase B (FatB) overexpression [17]. The acyl-ACP thioesterases, which terminate carbon chain elongation during FA biosynthesis, are classified into two types based on sequence homology: unsaturated oleoyl-ACP is the substrate for the FatA type and saturated acyl-ACPs are the substrates for the FatB type [18]. FatB is a major determinant of saturated FA synthesis in *Arabidopsis thaliana* (*Arabidopsis*), and the ratio of FatA/FatB determines the degree of FA saturation—a higher transcriptional level of FatA than FatB yields a higher production of unsaturated FAs [19,20].

*Cuphea* is a genus of the Lythraceae family with about 260 species of herbaceous perennials and small shrubs with distinct FA composition [21,22]. Predominant seed oils in *Cuphea* plants are saturated medium-chain FAs, which are composed of 6–14 carbon atoms. However, the seed oils produced in different *Cuphea* species are quite diverse. Depending on the species, seeds produce caprylic acid (8:0), capric acid (10:0), lauric acid (12:0), myristic acid (14:0), linoleic acid (18:2), or linolenic acid (18:3) as a dominant component of the seed oil. The major FA components in *Cuphea hookeriana* seed oil are 8:0 (50.2%) and 10:0 (25.4%) [21]. Embryo- and seed-specific expression of mRNA transcripts and clear substrate preference for 8:0 and 10:0 FAs showed that *ChFatB2* plays an important role in determining *C. hookeriana* seed oil composition [23]. For example, *ChFatB2* transgenic *B. napus* accumulated 11%, 27%, and 2% of 8:0, 10:0, and 12:0 FAs, respectively, and the total saturated FAs including 14:0, 16:0, and 18:0 was approximately 45%. Even though the major FA components in *C. hookeriana* seed oil are 8:0 (50.2%) and 10:0 (25.4%), *C. hookeriana* also has 16:0-ACP thioesterase (*ChFatB1*), which is expressed in leaves, roots, maturing seeds, and other organs [18,21]. Seed-specific transgenic expression of *ChFatB1* in *B. napus* led to high 16:0 FA accumulation in seed oil along with slight increases in 14:0, 18:0, and 20:0 content, resulting in 30% saturated FAs in the seeds [18]. In *ChFatB1*-expressing transgenic *B. napus*, 8:0 and 10:0 FAs were not detected, showing that ChFatB1 does not have specificity toward 8:0 or 10:0. The seed oil content of *C. paucipetala* is about 40.0% of the seed weight, and the total saturated FA content is approximately 94%, composed of 10:0 (89.0%), 12:0 (2.1%), 14:0 (0.9%), and 16:0 FAs (1.8%) [24]. Unsaturated FAs include 18:1 (1.5%) and 18:2 (3.7%) FAs.

Two cDNA clones, encoding *CpFatB4* from *C. paucipetala* (National Center for Biotechnology Information (NCBI) accession: AGL08247) and *CpFatB5* (NCBI accession: AGL08248) have been reported, but their biochemical activities have not yet been characterized. In addition, it has not been determined whether the expression of *B. napus* seed lipid metabolism genes, which can be further targeted for more desirable lipid traits, is affected by altered FA composition. In the present study, we generated transgenic *B. napus* overexpressing *C. paucipetala CpFatB4* or *CpFatB5* in a seed-specific manner and investigated (1) whether overexpression of each gene induced lipid composition changes in the seed, and if so, which FAs were affected, and (2) which genes had altered expression patterns in response to distinct lipid composition in different transgenic plants.

## 2. Results

### 2.1. Seed FA Profiles (mol%) and 100-Seed Weights Showed

#### Different Patterns Depending on the Transgene Expressed

Seed FA profiles were analyzed for non-transgenic *Youngsan* and transgenic plants overexpressing *CpFatB4* or *CpFatB5* (Table 1). Dry seeds of *CpFatB4* contained a higher ratio of saturated FAs—an average of 28.4% total FAs, ranging up to 31.0%. The percent content of each individual saturated FA species (14:0, 16:0, 18:0, and 20:0) was also increased, and that for 16:0 FA was most remarkable—about 4.3 times more than control. Dry seeds from *CpFatB5*-overexpressing plants also showed an increase in the total saturated FA content to 16.0%, but less than that of *CpFatB4*-overexpressing plants. However, a significant amount of 10:0 and 12:0 medium-chain FAs, which were not detected in the control, were observed in *CpFatB5* transgenic plants along with a higher amount of 16:0 FA than that in the control. For both transgenic plants, the FA composition changes were accompanied by the decrease in 18:1 FA. Among the three genotypes, the highest values for saturated FAs (14:0, 16:0, 18:0, and 20:0) and the lowest values for most unsaturated FAs (18:1, 18:2, 18:3, and 20:1) were found in *CpFatB4* seeds. Individual values for *CpFatB5* seeds were intermediate among the three plants, except for 10:0 and 12:0 FAs (Table 1). One-hundred seed weight was increased for *CpFatB4* seeds by 10.0%, whereas a 5% decrease was found for *CpFatB5* seeds.

### 2.2. Transcriptome Data Summary

**Figure 1:**
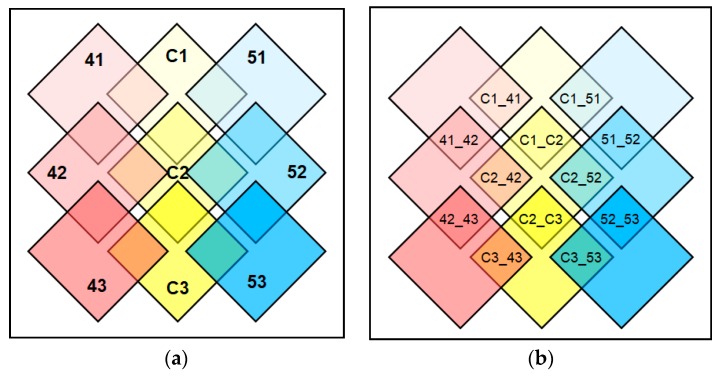
A diagram explaining how the RNA-Seq results were paired and compared to select differentially expressed genes (DEGs). (**a**) Large rectangles indicated by C1, C2, and C3 represent samples in 8 days after flowering (DAF), 25 DAF, and 45 DAF of *Youngsan* control plants, respectively. In the same manner, 41, 42, and 43 represent samples from 8 DAF, 25 DAF and 45 DAF of *CpFatB4*, while 51, 52, and 53 for *CpFatB5* transgenic plants in different stages in the order. (**b**) DEGs were identified in six pairs based on developmental stage and in another six pairs based on an overexpressed transgene, among nine samples in Figure 1a. The small rectangle made by the overlap of two neighboring large rectangles in Figure 1a shows DEGs between two samples, indicated by two large rectangles. For example, a small rectangle designated with 41_42 indicates DEGs detected by the log2-fold change between samples 41 and 42, and C1_41 indicates DEGs between samples C1 and 41.

For the RNA-Seq analysis, nine RNA samples of whole pods including developing seeds were used to generate RNA-Seq reads (Figure 1a). The nine samples used in our experiment were designated as C1 (8 days after flowering (DAF)), C2 (25 DAF), and C3 (45 DAF) for *Youngsan*, 41 (8 DAF), 42 (25 DAF), and 43 (45 DAF) for *CpFatB4* transgenic plants, and 51 (8 DAF), 52 (25 DAF), and 53 (45 DAF) for *CpFatB5* transgenic plants. In *Youngsan*, the accumulation of 18:1 linoleic acid, the predominant FA in this species, begins before 25 DAF [10]. In addition, the transcription of fatty acid biosynthesis genes is active between 2 and 6 WAP (weeks after pollination), and degradation dominates after 6 WAP in *B. napus* [13].

The RNA-Seq experiment generated a total of 286,854,290 clean reads, corresponding to the total length of 25,996,358,605 bp (Supplementary Table S1), after trimming the low-quality bases and removing reads shorter than 25 bp. A total of 91,830 transcripts were mapped onto the reference coding sequences (CDSs) (Supplementary Table S2), and the average mapping rate was 73.1%. Among the mapped transcripts, 87,513 genes had similar sequences (BLASTP: e-value ≤ e^−10^) in other plant species available at ARAPORT and in the Phytozome database [25,26]. Out of the annotated 87,513 genes, 47,312 (54.1%) and 34,219 (39.1%) matched those in *B. rapa* FPsc v1.3 and *B. rapa* Chiifu-401 v1.2, respectively. *B. rapa* is one of the progenitors of allotetraploid *B. napus*, and therefore both *B. rapa* and *B. napus* contain A genomes in the genus *Brassica* [8].

### 2.3. CpFatB4 and CpFatB5 Were Differentially Expressed during Seed Development

**Figure 2:**
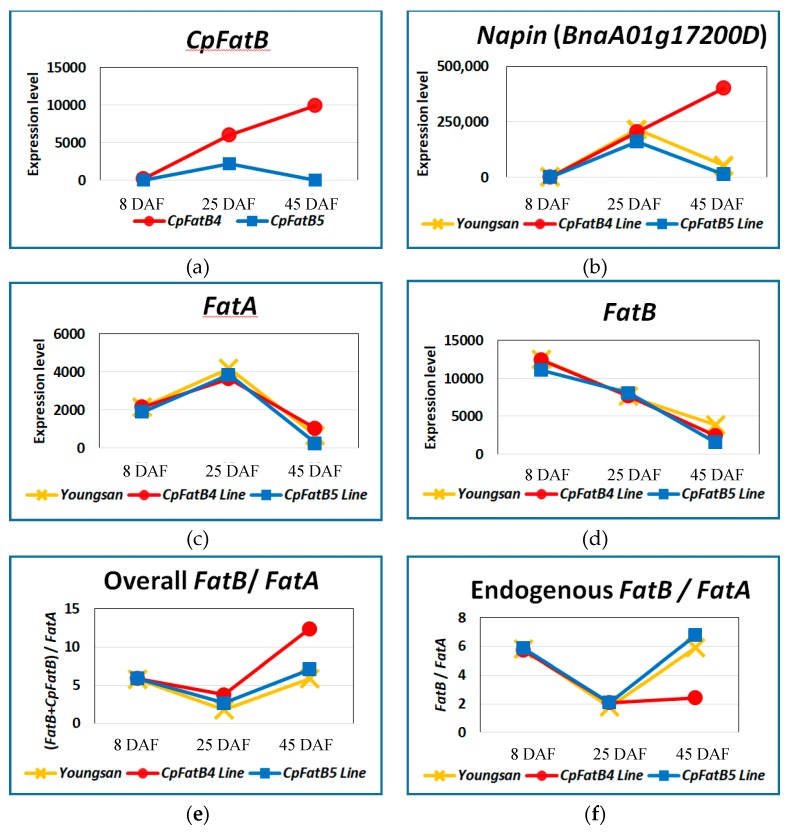
*Thioesterase* and *Napin* expression showed distinct patterns in *CpFatB4* and *CpFatB5* transgenic plants. (**a**) *CpFatB* expression in the corresponding transgenic plants. (**b**) Expression levels of *BnaA01g17200D*, a *Napin* gene, during pod development. (**c**) Sum of all endogenous *FatA* expression. (**d**) Sum of all endogenous *FatB* expression. (**e**) Ratio of overall expression levels of *FatA* and *FatB*, including transgenic *CpFatBs*. (**f**) Ratio of endogenous *FatB* and *FatA* expression in (**c**,**d**). Expression level was plotted using raw read numbers. *CpFatB*: read numbers of *CpFatB4* or *CpFatB5*; *CpFatB4* line: *CpFatB4* transgenic plant; *CpFatB5* line: *CpFatB5* transgenic plant; *FatA*: total read numbers of *FatA*; *FatB*: total read numbers of *FatB*; *Youngsan*: wild-type control.

Read numbers of *CpFatB4* or *CpFatB5* transcripts were compared among the samples to determine the expression levels and patterns. *CpFatB4* expression in *CpFatB4* transgenic plant was about 3 and 150 times higher than *CpFatB5* expression in the *CpFatB5* transgenic plant at 25 and 45 DAF, respectively, although these samples had comparable numbers of raw and clean reads (Figure 2a). The higher transcript levels and/or a continuous increase in the transgene expression detected in *CpFatB4* plants might be related to the more dramatic differences in FA composition, especially that for 16:0 FA, between *CpFatB4* transgenic plants and *CpFatB5* plants (Table 1 and Figure 2a).

### 2.4. CpFatB4 Expression under the Napin Promoter Affected Napin Promoter Activity

Different expression patterns were observed between *CpFatB4* and *CpFatB5* transcripts produced under the control of the same promoter. Among others, continuous increase in *CpFatB4* expression at 45 DAF was noticeable in *CpFatB4* transgenic plants, whereas *CpFatB5* expression at the same stage was lower than that of 25 DAF in *CpFatB5* transgenic plants (Figure 2a). Therefore, we examined whether this discrepancy was caused by the altered FA composition associated with *CpFatB4* transgene expression (Table 1) or simply by a position effect related to the genomic location of the transgene. We predicted that the transcriptional activity of endogenous *Napin* genes would also change in a manner similar to that of *CpFatB4* if the higher expression of *CpFatB4* transcripts at 45 DAF was caused by the altered lipid metabolism. The promoter sequence used in our experiment (NCBI accession: EU723261) corresponds to the sequence on chromosome C01 (13,718,611–13,719,745). Although no gene model was proposed in the reference genome [8], the downstream region of the proposed *Napin* promoter was transcriptionally active in our RNA samples and displayed almost perfect sequence identity with many reported *Napin* EST clones (NCBI accession: XM_013832756, FG578999, etc.). This finding confirmed that the promoter sequence used in our experiments directed the expression of *Napin* transcripts.

Many *Napin* genes showed altered expression patterns in the later stages of *CpFatB4* transgenic plants similar to those of *CpFatB4*. *Napin* genes encode storage proteins that belong to the 2S albumin family and are the second most abundant protein (20% of total protein) in *B. napus* seeds, following cruciferin (60%) [27]. *Napin* gene expression was high at stages 2 and 3, such as that of *BnaC01g43250D*, *BnaC01g19320D*, and *BnaA01g17200D* (Supplementary Table S2). In *CpFatB4* transgenic plants, expression level for all *Napin* genes—including *BnaA01g17200D*—was even higher at stage 3 than at stage 2 (Figure 2b). The nucleotide sequence of *BnaA01g17200D* was almost identical to that of the unannotated *Napin* gene on chromosome C01 described above, but originated from another progenitor of *B. napus*: *B. oleracea*. In addition to *Napin* genes, the mRNA expression levels for another type of storage protein genes, *Cruciferins*, such as *BnaA01g08350D* and *BnaC05g02160D*, were also higher in *CpFatB4* transgenic plants than in other plants when expression levels between 25 and 45 DAF were compared (Supplementary Table S2). The promoter behaviors of *Napin* and some other genes at 45 DAF might have changed in *CpFatB4* transgenic plants due to the FA content change.

### 2.5. CpFatB4 Expression Resulted in an Increase in the Overall FatB/FatA Ratio, but a Clear Decrease in Endogenous FatB/FatA Ratio

*CpFatB4* expression under the *Napin* promoter resulted in relatively small changes in the total expression levels of endogenous *FatA* or *FatB* genes. Expression levels of *B. napus* thioesterases were monitored to determine whether heterologous overexpression of a *C. paucipetala* acyl-ACP thioesterase affected the expression of endogenous genes with similar functions. In the *B. napus* genome, six *FatA* and six *FatB* genes were annotated based on their sequence similarity to functionally characterized *Arabidopsis* genes and synteny [8]: *BnaA03g37700D*, *BnaC03g74210D*, *BnaA07g05070D*, *BnaCnng00070D*, *BnaA04g07120D*, and *BnaCnng41490D* as *FatA* and *BnaA06g04900D*, *BnaC05g06160D*, *BnaA08g26890D*, *BnaC08g13600D*, *BnaAnng26510D*, and *BnaC08g43130D* as *FatB*. In addition to these 12 genes, we found that *BnaC06g08830D* and *BnaA10g09300D* also showed sequence similarity to *Arabidopsis FatA* (*At3g25110*) and *FatB* (*At1g08510*), respectively. Among these, *FatA BnaCnng41490D* and *FatB BnaA08g26890D* have previously been functionally characterized [17,28]. The expression levels for the *FatA*s and *FatB*s as a whole were generally similar at all three developmental stages between the three lines, although a slight increase of endogenous *FatA* expression in *CpFatB4* and a small decrease of endogenous *FatB* expression in *CpFatB4* and *CpFatB5* lines were observed at 45 DAF (Figure 2c,d).

We found that the overall *FatB*/*FatA* ratio was higher in *CpFatB4* transgenic plants than in *Youngsan* at 45 DAF owing to *CpFatB4* expression (Figure 2e). Previously, it was proposed that the ratio of *FatB*/*FatA* determines the degree of FA saturation, and higher expression level of *FatB* than *FatA* leads to a greater production of saturated FAs [20]. When only endogenous gene expression was considered, however, a much lower *FatB*/*FatA* ratio was observed at 45 DAF in the *CpFatB4* line compared to control and *CpFatB5* lines, owing to a small increase in *FatA*s and a small decrease in *FatB*s (Figure 2f). This observation indicated that *CpFatB4* transgenic plants contained higher saturated FA content owing to the overexpression of *CpFatB4*, especially at 45 DAF. In contrast to *CpFatB4* transgenic plants, no clear overall and endogenous *FatB*/*FatA* ratio changes were observed in *CpFatB5* transgenic plants (Figure 2e,f).

### 2.6. Depending on Developmental Stages or Genotypes, the Expressed Genes Showed Overlapping yet Distinct Expression Patterns

**Figure 3:**
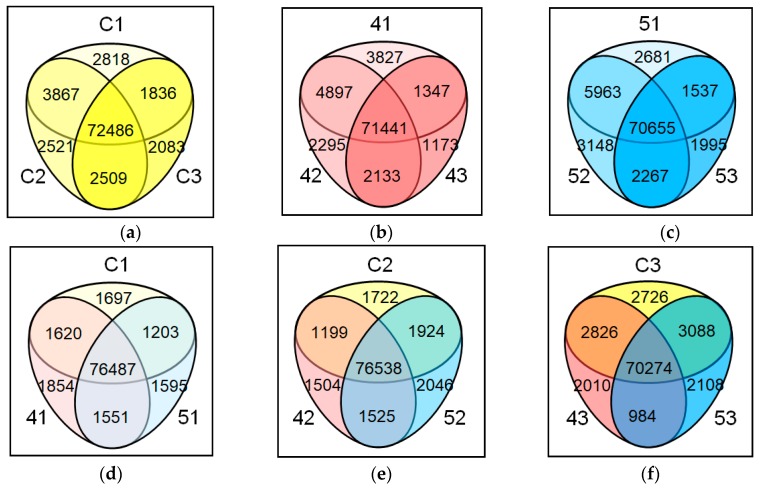
Venn diagrams for the expressed gene numbers showing overlapping yet distinct expression patterns depending on developmental stages or genotypes. (**a**) *Youngsan* at three different stages (8, 25, and 45 DAF): C1, C2, and C3. (**b**) *CpFatB4* transgenic line at three different stages: 41, 42, and 43. (**c**) *CpFatB5* transgenic line at three different stages: 51, 52, and 53. (**d**) *Youngsan*, *CpFatB4*, and *CpFatB5* at 8 DAF: C1, 41, and 51. (**e**) *Youngsan*, *CpFatB4*, and *CpFatB5* at 25 DAF: C2, 42, and 52. (**f**) *Youngsan*, *CpFatB4*, and *CpFatB5* at 45 DAF: C3, 43, and 53.

When the expressed genes were compared between different stages or genotypes, large numbers of genes were commonly expressed in all conditions considered. For example, 72,486 genes out of a total 101,040 genes in *B. napus* were commonly expressed in all three developmental stages of *Youngsan* (Figure 3a). Similar levels of overlaps—71,441 and 70,655 genes, respectively—were also observed in *CpFatB4* and *CpFatB5* transgenic lines (Figure 3b,c). Among the three lines with different genotypes, over seventy-thousand overlapping genes (76,487, 76,538, and 70,274, respectively) were identified at 8, 25, and 45 DAF (Figure 3d–f). At 45 DAF, a higher degree of non-overlapping gene expressions between genotypes was identified, reflecting gene expression changes associated with different seed FA compositions in each line (Table 1 and Figure 3f): over two thousand genes (2726, 2010, and 2108, respectively) were exclusively detected in “C3”, “43”, and “53”.

Differentially expressed genes (DEGs) between growth stages or different genotypes reflected gene activity changes. The differential expression levels of the genes were determined by pairwise comparisons, as described in Figure 1b. The numbers of DEGs are summarized in Table 2 and the DEGs are listed in Supplementary Table S2. The stage-comparisons among *Youngsan* (C1_C2 and C2_ C3), *CpFatB4* (41_42 and 42_43), and *CpFatB5* (51_52 and 52_53) lines revealed that there were significantly lower total DEG numbers in 42_43 (2,445 genes) than in C2_C3 (4,642 genes) or 52_53 (4,813 genes). In contrast, C1_C2 (2550 genes), 41_42 (2486 genes), and 51_52 (2575 genes) had similar numbers of total DEGs. While the number of up-regulated genes was 15% more than that of down-regulated genes in C2_C3, the number of down-regulated genes was 38% and 75% more than that of up-regulated genes in 42_43 and 52_53, respectively. These results indicated that the heterologous expression of *CpFatB4* or *CpFatB5* genes had transcriptome-wide effects on gene expression at later stages, and the effect was also obvious in *CpFatB5* plants with relatively minor FA changes (Table 1). DEG analyses between different lines at the same developmental stage (e.g., C1_41, C2_42, C3_43, C1_51, C2_52, and C3_53) revealed much lower numbers of DEGs in C1_41 (366 genes), C1_51 (289 genes), C2_42 (45 genes), and C2_52 (112 genes) than between-stage comparisons for a given genotype. These results indicated that gene activity changes between control and transgenic plants were more obvious at later developmental stages.

The ratio of lipid metabolism DEGs to total DEGs was highest (12.3%) in up-regulated genes in the C3_43 comparison (Table 2). Especially, almost half of all lipid metabolism DEGs in genotype comparisons were up-regulated in C3_43 (83 out of 175 genes). Even when both up- and down-regulated DEGs were taken together, lipid metabolism genes showed the most significant differences in C3_43 (105 out of 175 genes). However, no DEG for lipid-related genes was observed in C2_42, suggesting that lipid metabolism was very similar between the *Youngsan* and *CpFatB4* lines at 25 DAF. The complete list of lipid metabolism genes in *B. napus* and their expression changes are summarized in Supplementary Table S4.

### 2.7. Among the Top 20 DEGs, Similarities Were Found in DEGs by Developmental Stages, but No Common Gene Was Identified in DEGs by Genotypes

The top 20 genes showing the strongest differential expression in each comparison are listed and annotated based on their sequence similarities to *Arabidopsis* genes in Supplementary Table S5. Some common DEGs were identified in all three lines despite their genotypic differences from stage comparisons in each line. These DEGs included genes for (1) the RmlC-like cupin superfamily proteins and laccase/diphenol oxidase family proteins in a comparison between 8 and 25 DAF and (2) late embryogenesis abundant protein (LEA) protein M10 and mannose-binding lectin superfamily proteins between 25 and 45 DAF. Some top-20 DEGs in a given genotype were found among the top 20 DEGs in only two genotypes or were exclusively present in one line, but most of them were still detected as DEGs in other genotypes but below the top 20. For example, *3-ketoacyl-CoA synthase 18* and *Cruciferin* were found among DEGs in C1_C2 and 41_42 comparisons, and genes for LEA-domain-containing protein and cupin family protein were identified as DEGs in the C2_C3 and 52_53 comparisons.

In contrast, no common gene was identified among the top 20 DEGs when the *CpFatB4* or *CpFatB5* lines were compared with *Youngsan* at each stage (Supplementary Table S6). Nonetheless, *cold, circadian rhythm, and RNA binding 2* was identified as one of the top 20 DEGs comparing *CpFatB4* and *Youngsan* at 8 DAF (C1_41) and was also identified as a DEG ranked below 20 in the C1_51 comparison. The C2_42 comparison at 25 DAF showed that genes for RmlC-like cupin superfamily proteins, galactose oxidase/kelch repeat superfamily protein, and cruciferin 3 were up-regulated, while genes for photosystem II reaction center protein A were down-regulated among the top 20 DEGs. In the C2_52 comparison, up-regulation of *photosystem I*, *PsaA/PsaB protein*, *photosystem II reaction center protein C*, and *ribulose-bisphosphate carboxylases* was detected. At 45 DAF, the top 20 DEGs in C3_43 contained the genes for seed storage albumin 3,2-oxoglutarate (2OG) and Fe(II)-dependent oxygenase superfamily protein, GDSL-like lipase/acyl-hydrolase superfamily protein, and photosystem II reaction center protein A as up-regulated genes, and those for P-loop-containing nucleoside triphosphate hydrolases superfamily protein and zincin-like metalloproteases family protein as down-regulated genes.

### 2.8. Gene Ontology (GO) and Kyoto Encyclopedia of Genes and Genome (KEGG) Analyses of DEGs Showed Gene Enrichment in Some Categories, Such as “Fatty Acid Biosynthetic Process” and “Glycolysis”, in CpFatB4 at 45 DAF

**Figure 4:**
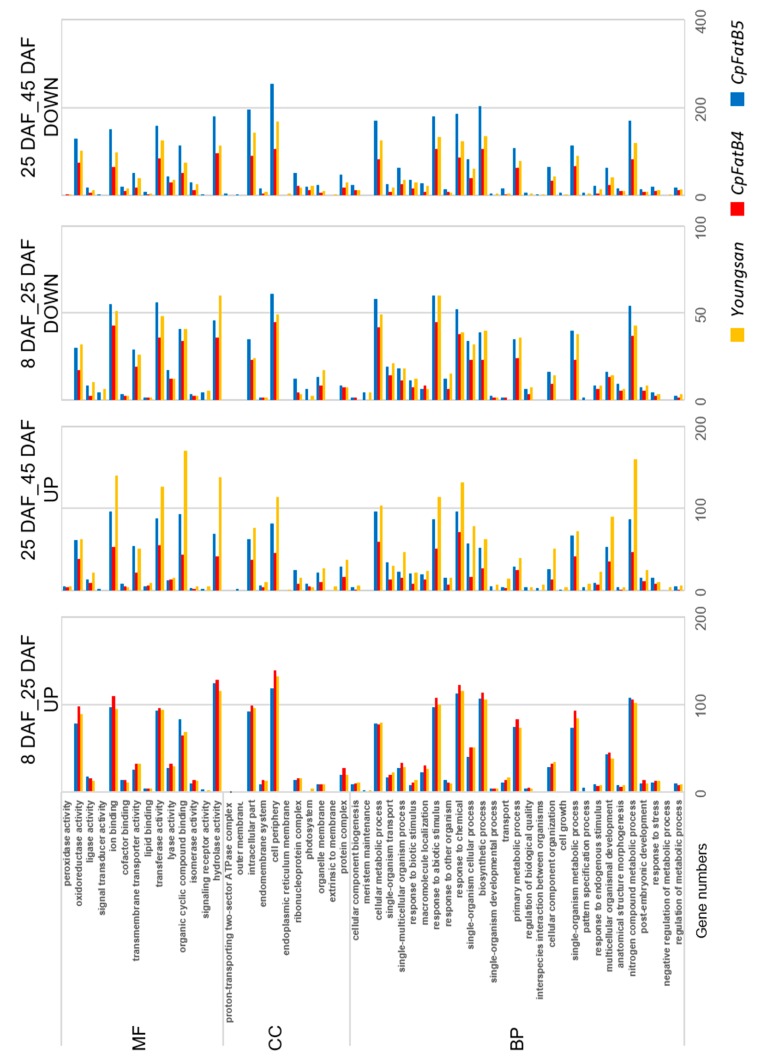
Numbers of DEGs by developmental stages in different genotypes based on Gene Ontology (GO) terms. DEG numbers determined from comparison between developmental stages were plotted according to GO terms. 8 DAF_25 DAF: comparison between 8 and 25 DAF; 25 DAF_45 DAF: comparison between 25 and 45 DAF; BP: biological process; CC: cellular component; DOWN: down-regulated; MF: molecular function; UP: up-regulated.

Among the 87,513 annotated genes in this study, 51,026 genes were assigned with GO IDs (Supplementary Table S2). DEGs in each genotype by growth stage were classified into 54 GO terms: 29, 11, and 14 GO IDs in biological process (BP), cellular component (CC), and molecular function (MF), respectively (Supplementary Table S7). In the C3_43 comparison, many up-regulated DEGs were found in the response to abiotic stimulus, response to chemical, biosynthetic process, primary metabolic process, intracellular part, cell periphery, ion binding, transferase activity, and hydrolase activity categories. When 676 up-regulated DEGs in C3_43 (Table 2) were analyzed for enrichment, glycolysis (*p*-value: 8.71 × 10^−37^, false discovery rate (FDR): 2.85 × 10^−34^), oxidation-reduction process (*p*-value: 5.76 × 10^−34^, FDR: 9.4 × 10^−32^), fatty acid biosynthetic process (*p*-value: 3.71 × 10^−23^, FDR: 4.04 × 10^−21^), and fatty acid metabolic process (*p*-value: 6.30 × 10^−7^, FDR: 1.36 × 10^−5^) were found to be the most enriched in the GO BP category (Supplementary Table S8). In addition, enrichment of nutrient reservoir activity, catalytic activity, magnesium ion binding, thylakoid, photosystem, and photosystem I in MF and CC GO categories were observed. For 1171 down-regulated DEGs in the C3_43 comparison, nucleic acid binding, nucleotide binding, RNA processing, and small-subunit processome GO categories were enriched. When GO term enrichment was examined for up-regulated DEGs between C3 and 53 samples, it was found that glycolysis and fatty acid biosynthetic process as well as nutrient reservoir activity categories were enriched only in *CpFatB4*, but not in C3_53. Nutrient reservoir activity and fatty acid metabolic process—two enriched GO categories with up-regulated DEGs in C3_43—were found among the down-regulated DEGs in C3_53 (Supplementary Table S8). Owing to the small numbers of DEGs detected in the C2_42 comparison, no over-representation was found in this comparison. Distinct GO enrichment patterns were observed between developmental stages in three genotypes (Figure 4). When expression at 25 and 45 DAF were compared, DEG numbers for up-regulated genes were generally larger in *Youngsan* than in other genotypes, whereas those for down-regulated genes were usually larger in *CpFatB5*. Up-regulated DEG numbers between 8 and 25 DAF were slightly larger in *CpFatB4* plants than in other genotypes, but smaller numbers of DEGs were detected among up- or down-regulated DEGs between 25 and 45 DAF (Figure 4). The distinct patterns observed in GO categories for *CpFatB4* DEGs might be related to a clear difference in saturated FA content in *CpFatB4* seeds.

In our study, 15,626 *B. napus* genes were annotated with KO identifiers [29]. These genes are located in 369 pathways, based on the KEGG pathway database (Supplementary Table S9). The influence on gene expression by either transgene was identified using Reconstruct Pathway. Among others, comparisons between *Youngsan* and transgenic plants at 45 DAF, C3_43, and C3_53 showed that more dynamic cellular adjustments occurred in the *CpFatB4* transgenic line at this stage (Supplementary Table S9). In the fatty acid metabolism pathway among global and overview maps category, there were nine up-regulated DEGs in C3_43, but none in C3_53. More specifically, fatty acid biosynthesis, fatty acid elongation, fatty acid degradation, steroid biosynthesis, and biosynthesis of unsaturated fatty acids pathways in the lipid metabolism category were represented by up-regulated DEGs in C3_43, whereas no DEGs were detected in C1_41, C2_42, C1_51, C2_52, and C3_53. In C3_43, several up-regulated DEGs were found in the carbohydrate metabolism and biosynthesis of other secondary metabolites as well as folding, sorting, and degradation pathways, but usually none in C3_53. In the replication and repair pathway in the genetic information processing category, down-regulated DEGs were also specifically identified in C3_43, with respect to C3_53.

### 2.9. Lipid Metabolism DEGs with the Strongest Expression Changes Were Different between CpFatB4 and CpFatB5

Among *B. napus* lipid metabolism DEGs, the top 20 DEGs showing the highest levels of differential expression (up or down) were identified in each between-stage or between-line comparison (Supplementary Table S10). *B. napus* lipid metabolism genes were previously reported based on sequence similarity and synteny with *Arabidopsis* lipid-related genes listed in the ARALIP database [8]. The numbers of lipid-related DEGs in each comparison are shown in Table 2. In all comparisons between 8 and 25 DAF, C1_C2, 41_42, and 51_52, *ketoacyl-CoA synthase*, *glucose-methanol-choline oxidoreductase family protein*, *oil-body oleosin*, *homologs of maize transcription factor Viviparous-1*, and *SAD* were included among the top 20 up-regulated lipid metabolism DEGs (Supplementary Table S10). In comparisons between 25 and 45 DAF, C2_C3, 42_43, and 52_53, steroleosin and *acyl-hydrolase* (*patatin-like*) were the common top 20 up-regulated DEGs. *Alcohol-forming fatty acyl-CoA reductase* and *lipid transfer protein* were in the common top 20 down-regulated DEGs. Most of the other lipid metabolism DEGs included as the top 20 in only one or two comparisons were found in the DEG lists, but below top 20. Such DEGs included *caleosin* in C1_C2 and 41_42, *long-chain acyl-CoA synthetase* in C2_C3 and 52_53, and *phospholipase C* in 51_52 as up-regulated, whereas *SAD* in C2_C3 and 52_53 and *ketoacyl-CoA thiolase* in C2_C3 as down-regulated.

When compared to *Youngsan* control, *CpFatB4* and *CpFatB5* lines showed a few common lipid metabolism DEGs, such as the up-regulated *carboxyltransferase beta subunit of heteromeric acetyl CoA carboxylase* (*ACCase*) and *ketoacyl-CoA synthase* at 45 DAF and down-regulated *myo-inositol-3-phosphate synthase* at 8 DAF (Supplementary Table S10). In C3_43, *triacylglycerol* (*TAG*) *lipase*, *linoleate desaturase*, *HXXXD-type acyl-transferase family protein*, *SAD*, and *phosphatidylinositol-4-kinase gamma*, along with the common DEGs mentioned above, were the top 20 lipid-related DEGs. While up-regulated in C3_43, *pyruvate dehydrogenase alpha subunit* and *ketoacyl-CoA synthase* were found in the top 20 down-regulated DEGs in C3_53, consistent with distinct lipid metabolisms induced by *CpFatB4* or *CpFatB5* transgene (Supplementary Tables S4 and S10).

### 2.10. Plastidial FA Synthesis Pathway Was Activated by CpFatB4 Overexpression, but TAG Synthesis Was Not Strongly Affected

**Figure 5:**
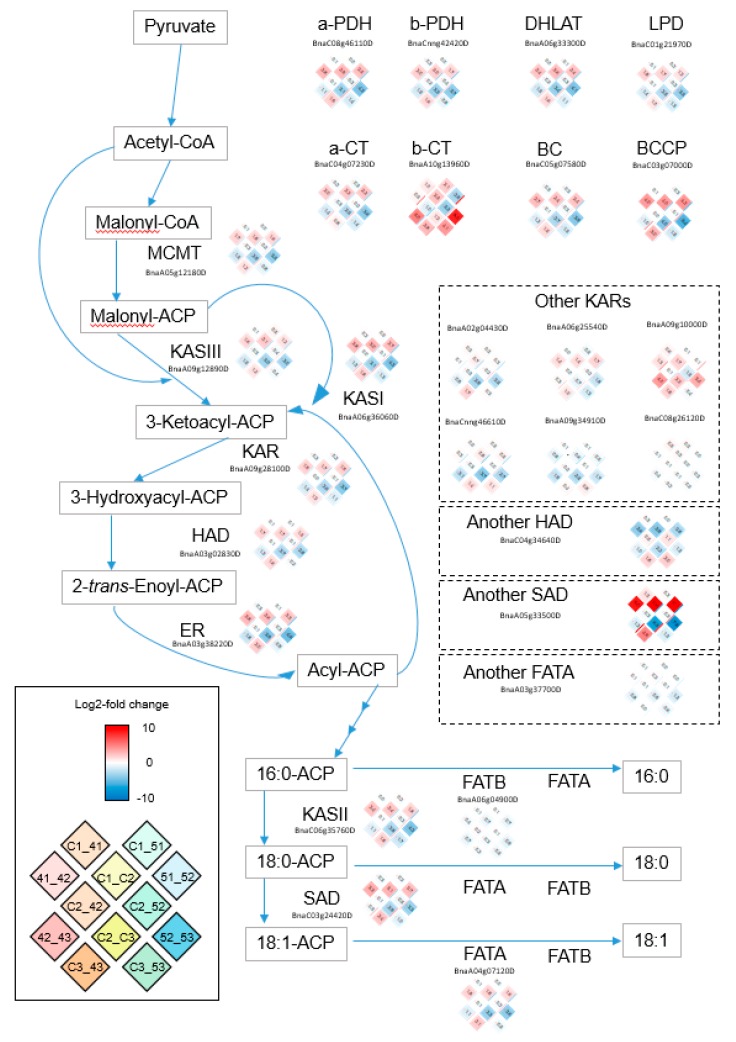
*B. napus* genes involved in the plastidial fatty acid synthesis and their log2-fold expression changes. As representatives of genes involved in the same biochemical step, log2-fold changes of the most highly expressed genes are shown: the expression patterns were similar among them, except those stated separately and shown in the dotted boxes. Solid box: heatmap scale of log2-fold gene expression change and positions of log2-fold change for 12 pair-wise comparisons. α-PDH: pyruvate dehydrogenase alpha subunit; α-CT: carboxyltransferase alpha subunit of heteromeric ACCase; β-CT: carboxyltransferase beta subunit of heteromeric ACCase; β-PDH: pyruvate dehydrogenase beta subunit; BC: biotin carboxylase, a subunit of heteromeric ACCase; BCCP: biotin carboxyl carrier protein, a subunit of heteromeric ACCase; DHLAT: dihydrolipoamide acetyltransferase; ER: enoyl-ACP reductase; FAT: acyl-ACP thioesterase; HAD: hydroxy acyl-ACP dehydrase; KAR: ketoacyl-ACP reductase; KAS: ketoacyl-ACP synthase; LPD: dihydrolipoamide dehydrogenase; MCMT: malonyl-CoA:ACP malonyltransferase; SAD: stearoyl-ACP desaturase.

**Figure 6:**
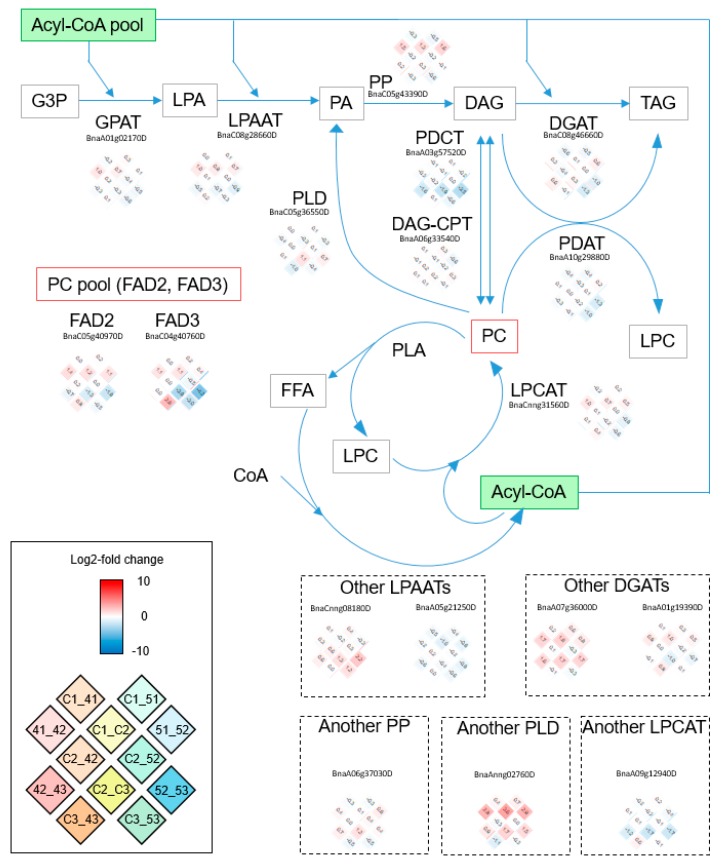
*B. napus* genes involved in triacylglycerol (TAG) synthesis and their log2-fold expression changes. As representatives of genes involved in the same biochemical step, log2-fold changes of the most highly expressed genes are shown: the expression patterns were similar among them, except those stated separately. Those showing somewhat different expression patterns are indicated in dotted boxes. Solid box: heatmap scale of log2-fold gene expression change and positions of log2-fold change for 12 pair-wise comparisons. DAG: 1,2-diacylglycerol; DGAT: acyl-CoA:diacylglycerol acyltransferase; DAG-CPT: diacylglycerol cholinephosphotransferase; FAD2: oleate desaturase; FAD3: linoleate desaturase; FFA: free fatty acid; G3P: glycerol-3-phosphate; GPAT: glycerol-3-phosphate acyltransferase; LPA: lysophosphatidic acid; LPAAT: lysophosphatidic acid acyltransferase; LPC: lysophosphatidylcholine; LPCAT: lysophosphosphatidylcholine acyltransferase; PA: 1,2-diacylglycerol-3-phosphate; PC: phosphatidylcholine; PDAT: phospholipid:diacylglycerol acyltransferase; PDCT: phosphatidylcholine:diacylglycerol cholinephosphotransferase; PLA: phospholipase A2; PLD: phospholipase D zeta; PP: phosphatidate phosphatase.

Many plastidial FA synthesis genes were up-regulated in the C3_43 comparison when all the lipid-related DEGs were examined (Supplementary Table S4). Figure 5 describes the plastidial FA synthesis pathway, and log2-fold changes in gene expressions are shown in the heat map for all corresponding lipid-related genes detected in our RNA-Seq analyses. Except for *FatB*, all genes in Figure 4 were more strongly expressed in *CpFatB4* than in *Youngsan* (as indicated in the lower-left corner of the heat map for each gene). In contrast, the genes involved in the TAG synthesis pathway were not noticeably up- or down-regulated in C3_43 (Figure 6), indicating that the TAG synthesis pathway was not considerably disturbed by the overexpression of *CpFatB4*. Genes involved in the same biochemical step showed similar expression patterns in log2-fold changes. However, complex expression patterns were observed for *KAR* (*ketoacyl-ACP reductase*), *LPAAT*, and other enzymes (Figure 5 and Figure 6). The genes for FA unsaturation, such as *SAD* in Figure 5 and *FAD2* and *FAD3* in Figure 6, were up-regulated in C3_43. This change might be induced to compensate for the reduction of unsaturated FAs in *CpFatB4*.

Among lipid metabolism DEGs, up-regulated genes were notable in *CpFatB4* when compared to *Youngsan* control, whereas many down-regulated genes were observed in *CpFatB5*. In C3_43, *β-Ketoacyl-acyl carrier protein synthase* (*KAS*) *I*, *KAS II*, *SAD*, *FAD5-like desaturase*, *oleate desaturase*, and *linoleate desaturase* were identified as up-regulated lipid-related DEGs (Figure 5 and Supplementary Table S4). Some down-regulated DEGs in C3_53 included *SADs*, *phospholipid:diacylglycerol acyltransferase* (*PDAT*), *acyl-CoA:diacylglycerol acyltransferase* (*DGAT*), *acyl-CoA oxidase*, and *ketoacyl-CoA synthase* (Figure 5 and Figure 6, Supplementary Table S4). The only up-regulated lipid-related DEG in the C3_53 was the plastid-encoded *carboxyltransferase beta subunit of heteromeric ACCase*. Other genes encoding nuclear-encoded ACCase subunits such as *carboxyltransferase alpha subunit*, *biotin carboxylase*, and *biotin carboxyl carrier protein* were found to be down-regulated in C3_53 (Figure 5 and Supplementary Table S4).

### 2.11. RNA-Seq Results Were Validated by RT-qPCR

**Figure 7:**
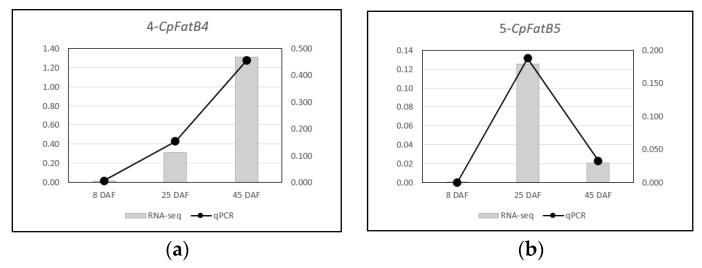
Comparison of RNA-Seq and RT-qPCR expressions of *CpFatB4* and *CpFatB5* transgenes. (**a**) 4-CpFatB4: *CpFatB4* gene expression in developing pods of *CpFatB4* transgenic plant. (**b**) 5-CpFatB5: *CpFatB5* gene expression in developing pods of *CpFatB5* transgenic plant. Primary axis (left vertical axis) values are for RNA-Seq and secondary axis (right vertical axis) values are for RT-qPCR.

**Figure 8:**
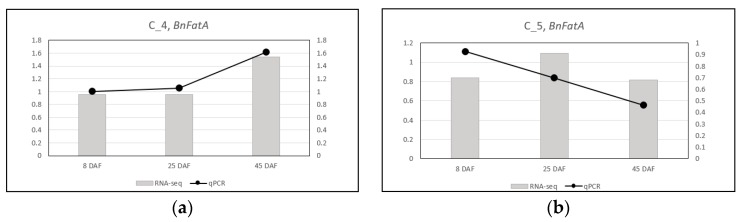

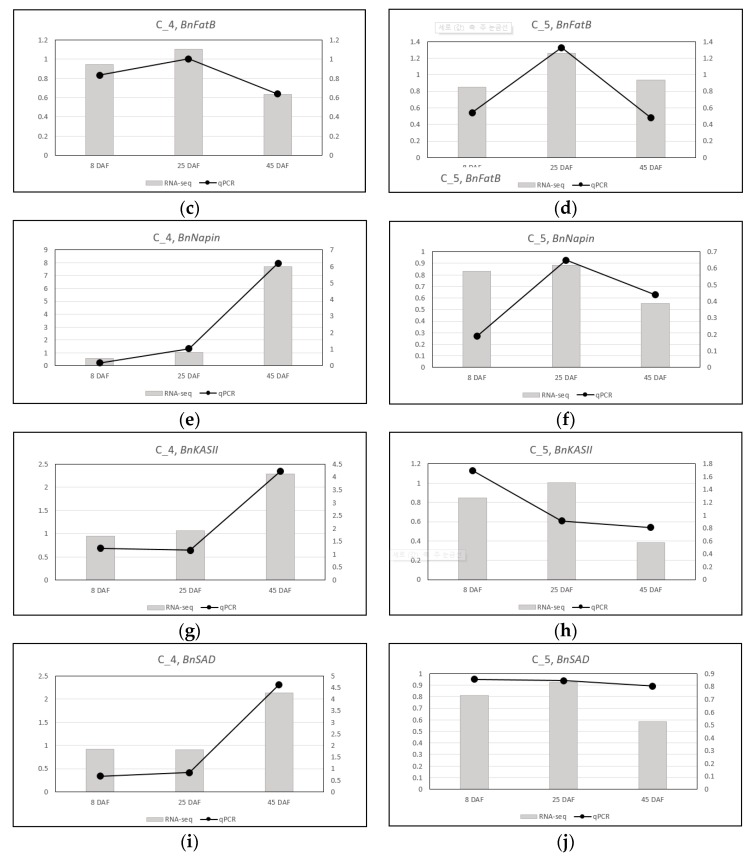
Comparison of RNA-Seq and RT-qPCR expressions of endogenous genes in *CpFatB4* and *CpFatB5* transgenic plants. (**a**,**c**,**e**,**g**,**i**) Expression changes of *B. napus FatA* (*BnFatA*), *FatB* (*BnFatB*), *Napin* (*BnNapin*), *KASII* (*BnKASII*), and *SAD* (*BnSAD*) genes in *CpFatB4*, compared to control plants (C_4). (**b**,**d**,**f**,**h**,**j**) Expression changes of *B. napus FatA* (*BnFatA*), *FatB* (*BnFatB*), *Napin* (*BnNapin*), *KASII* (*BnKASII*), and *SAD* (*BnSAD*) genes in *CpFatB5*, compared to control plants (C_5). Primary axis (left vertical axis) values are for RNA-Seq and secondary axis (right vertical axis) values are for RT-qPCR.

After normalizing C_T_ values from the RT-qPCR results relative to those of *β-Actin*, RT-qPCR results were compared with the modified RNA-Seq results in which read numbers for each gene were divided by those of *β-Actin*. We found that there were high levels of correlation between RNA-Seq and qPCR results for *CpFatB4* and *CpFatB5* expression in developing pods of corresponding transgenic plants, showing R-squared values of 0.9905 and 0.9999, respectively (Figure 7 and Figure 8). Similar to RNA-Seq results in Figure 2A, the *CpFatB4* expression level continuously increased throughout the seed development, while *CpFatB5* expression did not (Figure 7). We also observed comparable trends between RNA-Seq and RT-qPCR, when *β-Actin* normalized values of *B. napus FatA, FatB, Napin, KASII*, and *SAD* genes from the control line were used for relative quantification of those in *CpFatB4* and *CpFatB5* transgenic lines by RT-qPCR (Figure 5 and Figure 8). Among others, a good correlation of *Napin* expression changes in *CpFatB4* transgenic plant was evident (Figure 8E). Based on these observations, we concluded that expression levels by RT-qPCR support the RNA-Seq results well.

## 3. Discussion

The distinct biochemical preferences of *CpFatB4* and *CpFatB5* for palmitic acid and medium-chain FA biosynthesis were characterized using transgenic *B. napus*. *CpFatB4* has 95% identity with *ChFatB1* in *C. hookeriana*, which shows similar FA profiles when overexpressed in *B. napus*, particularly a more than 4-fold increase in 16:0 FA (Table 1) [18]. These results indicate that *CpFatB4* also preferably functions as 16:0-ACP thioesterase similar to *ChFatB1*. *CpFatB5* showed 96% sequence identity with *CvFatB1* in *C. viscosissima* [30]. *CvFatB1* mainly produces 8:0 (51 mol %) and 10:0 (25 mol %) FAs, based on FA profiles in *Escherichia coli* expressing CvFatB1. In this study using transgenic *B. napus*, *CpFatB5* expression also produced medium-chain FAs: 10:0 and 12:0 (Table 1). Taken together, we demonstrated that *CpFatB4* and *CpFatB5* function as thioesterases with different biochemical properties using the transgenic approach: the former is mainly involved in the production of 16:0 FA, whereas the latter produces shorter 10:0 and 12:0 FAs. The FA profiles in Table 1 show that the amount of each FA in *CpFatB5* was generally intermediate among the three genotypes compared, except for 10:0 and 12:0. *CpFatB4* had the highest values for saturated FAs (14:0, 16:0, 18:0, and 20:0) and the lowest values for most unsaturated FAs (18:1, 18:2, 18:3, and 20:1). One-hundred seed weight in *CpFatB4* transgenic plants showed about 10% increase to 250.1 mg from 227.4 mg of *Youngsan*, but *CpFatB5* showed approximately 5% decrease (Table 1). Further studies for the mechanism by which *CpFatB4* overexpression results in seed weight increase are needed.

### 3.1. Whole-Pod Transcriptomes in Transgenic B. napus Showed Similar Developmental Gene Expression Changes to Those of the Control

We analyzed the overall gene expression changes during seed development by selecting three time points (8, 25, and 45 DAF). Each growth stage could be characterized by the functions of the outstanding DEGs. The function of each DEG was predicted based on the sequence similarity to other plant genes using ThaleMine [25]. The strong up-regulation of many seed storage protein genes showed that seed storage protein started accumulating between 8 and 25 DAF: genes encoding RmlC-like cupin superfamily protein genes and seed storage albumin superfamily protein genes including *Napin* were identified among up-regulated DEGs. Compared to 25 DAF, the up-regulation of desiccation tolerance and maturation-related genes such as *LEA protein M10* or *stress-induced protein genes* indicated that these processes became evident at 45 DAF. DEGs identified by between-stage comparisons for each genotype showed that many DEGs were commonly found regardless of genotypes (Table S5). Although only a small number of the top 20 DEGs showing the strongest gene expression changes overlapped between three genotypes, most of the top 20 DEGs in a given genotype were still found among DEGs in the other genotypes when all DEGs were considered.

The expression patterns for FA biosynthesis genes were similar between seeds and entire pods of rapeseeds. In Brassicaceae plants, the pod wall is photosynthetically active and plays an important role in regulating seed growth and maturation [21,31,32]. Several previous studies have analyzed the *B. napus* transcriptome during seed development from 5 to 56 DAF, including one report using entire pods: (1) using seeds at 12–20, 21–25, 26–30, and 31–35 DAF [9]; (2) using seeds at 25 days after pollination (DAP) [12]; (3) using developing embryos at 17, 35, and 52 days after pollination [33]; (4) using pods at 5–7, 15–17, and 25–27 DAF [11]; and (5) using seeds at 2, 4, 6, and 8 weeks after pollination (WAP) [13]. Transcriptome analysis by Wan et al. reported the up-regulation of most of the genes involved in the FA biosynthesis pathway during 2 to 4 WAP [13], whereas Xu et al. showed only one up-regulated FA biosynthesis DEG across three stages [11]. Wan et al. suspected that this discrepancy might be related to different plant materials used in two studies: seeds or whole pods [13]. However, our analyses using whole pods showed that most genes involved in FA biosynthesis were up-regulated between 8 and 25 DAF, which was comparable to the results using seeds [13]. In our results, all representative FA biosynthesis genes showed “up-and-down” expression patterns during seed development in all genotypes; these are indicated by red colors (up-regulation) for comparisons between 8 and 25 DAF and blue colors (down-regulation) for comparisons between 25 and 45 DAF in Figure 5. Similar to our observation, bell-shaped temporal expressions for FA biosynthesis genes were reported during 2, 4, 6, and 8 WAP or 17, 35, and 52 DAP periods for developing embryos [13,33]. In the former, the highest expression was usually observed at 4 WAP. Note that the real expression levels of FA biosynthesis genes in the developing seeds changed more dramatically than those in our results using whole pods.

### 3.2. The Transcriptome Analyses Provided Comprehensive Gene Expression Changes Caused by 16:0 or 10:0/12:0 FA Accumulation in Seeds of B. napus

Lipid-related DEG changes occurred mostly at 45 DAF in transgenic lines. Lipids among the three genotypes used in our analysis were very similar at 8 and 25 DAF. Very small numbers of lipid-related DEGs (10, 6, and 6 genes) were identified in C1_41, C1_51, and C2_52 comparisons, respectively, and there was no DEG involved in lipid metabolism in C2_42 (Table 2 and Supplementary Table S4). Lipid-related DEGs in C3_43 were mostly up-regulated, whereas those in C3_53 were down-regulated. The only up-regulated DEG in C3_53 was the chloroplast-encoded gene *carboxyltransferase beta subunit of heteromeric ACCase*. In the C3_43 comparison, most of the plastidial FA synthesis-related genes including *KAS I*, *KAS II*, and desaturation genes (*stearoyl-ACP desaturase*, *FAD5-like desaturase*, *oleate desaturase*, and *linoleate desaturase*) were up-regulated (Figure 5). These gene expression changes in *CpFatB4* might be caused by the increase in palmitic acid and/or decrease in 18:1 oleic acid, and reflect gene expression reprogramming to compensate for altered fatty acid contents (Table 1).

A clear increase in overall *FatB*/*FatA* (*FatB* + *CpFatB*/*FatA*) ratio was observed at 45 DAF in the *CpFatB4* transgenic line (Figure 2E). A higher ratio of *FatA*/*FatB* is important to the degree of FA saturation, and leads to a greater production of unsaturated FAs [20]. In *B. napus*, *FatA* expression levels in seeds were higher than those of *FatB* [9,12], whereas *FatB* expression was higher than that of *FatA* in the leaves [12]. In our study using the whole pod, endogenous *FatB* expression levels were higher than *FatA* expressions, possibly due to the inclusion of pod walls in the sample (Figure 2F).

In *CpFatB4* transgenic plants, the expression of many storage protein genes including *Napin* was up-regulated, especially at 45 DAF. Prompted by the altered behavior of the *Napin* promoter used for the transgene expression of *CpFatB4* (Figure 2B, and Supplementary Table S2), we investigated further and found that the expressions of many *B. napus* endogenous storage protein genes showed a continuous increase at 45 DAF in *CpFatB4*, whereas their expression levels decreased in control and *CpFatB5* transgenic plants. These storage protein genes in *B. napus* encode both cruciferins and napins, which are major storage proteins and belonging to cupin and albumin superfamilies, respectively [27]. Given that *CpFatB5*, which was also expressed under the control of the same *Napin* promoter in *CpFatB5* transgenic plants, showed a rather decreased expression of storage protein genes compared to *Youngsan* control, we speculate that the increase in storage protein gene expression in *CpFatB4* was caused by increased 16:0 FA content and/or decreased 18:1 (Table 1 and Supplementary Table S2).

With a significant increase in 16:0, the FA profile of *CpFatB4* seeds is a step closer to that of palm oil. When combined with further genetic modifications (most importantly those reducing the amount of 18:1), *CpFatB4* overexpression in *B. napus* would provide one useful route to substitute palm oil in the non-tropical region. The silencing of *SAD*s, combined with the overexpression of a native *FatB*, resulted in an increase of 16:0 up to 31% [17], compared to about 23% in *CpFatB4* transgenic plants (Table 1). According to our gene expression profiles, other *B. napus SAD*s, in addition to previously identified *BnaA05g03490D* and *BnaC04g03030D*, were also actively transcribed in developing *B. napus* pods at 25 DAF, and these other *SAD*s provide putative additional targets for genetic modification to further increase 16:0 FA [34,35].

Altered seed FA contents in *B. napus* are associated with changes in enzyme activities involved in FA metabolism. The accumulation of the physiological concentration of oleic acid (18:1) in developing seed extracts inhibited ACCase activity [36], and the increase of laurate in seed triacylglycerols by lauryl (12:0)-ACP thioesterase was found to be the result of a coordinated induction of the fatty acid synthesis pathway [37]. Although transcript levels of ACCase subunits do not always correspond to the ACCase enzyme activity (suggesting post-translational regulation [38,39]), the identification of DEGs between *B. napus* seeds with high and low oleic acid contents indicated that *B. napus* genes involved in FA metabolism are modulated by seed FA contents [40]. Although the detection of FA metabolism DEGs was unsuccessful in transgenic *Arabidopsis* seeds which overexpressed *C. lanceolata FatB3* and accumulated significant amounts of 10:0 medium-chain fatty acids, it might result from the fact that less than 200 genes were investigated in the microarray experiment [41]. Our results based on *B. napus* whole-genome information and unbiased RNA-Seq approach report comprehensive gene expression changes caused by distinct seed FA contents in *CpFatB4* or *CpFatB5* transgenic plants. Similar approaches will facilitate the identification of target genes for further modification to establish transgenic plants with desired FA contents.

### 3.3. DEGs Detected in CpFatB5 Suggest the Modulation of Organellar Gene Expression Responding to Medium-Chain FA Accumulation

Many of the top 20 DEGs in the C2_52 comparison showed sequence similarity to *Arabidopsis* genes that are highly similar to *Arabidopsis* plastid-encoded genes, and most of the top 20 DEGs in C3_53 were similar to *Arabidopsis* mitochondria-encoded genes (Supplementary Table S6). Putative plastid-encoded or mitochondria-encoded DEGs were also detected in the *CpFat4* line, but to a lesser degree. When all DEGs at 45 DAF were considered, 180 and 31 *B. napus* DEGs, corresponding to 48 plastid-encoded and 21 mitochondria-encoded *Arabidopsis* genes, respectively, were identified in C3_53. Only 81 and 5 DEGs present in C3_43 were similar to *Arabidopsis* plastid-encoded and mitochondrial-encoded genes, respectively (Supplementary Table S2). The high proportions of putatively plastid- or mitochondria-encoded DEGs in C3_53 and C3_43 raises the possibility that the medium-chain FAs produced in the transgenic lines may function as signaling molecules to modulate the organellar gene expressions. Further studies on regulation mechanisms are required.

## 4. Materials and Methods

### 4.1. Plant Materials

Transgenic *B. napus* lines expressing *C. paucipetala* acyl-ACP thioesterase *CpFatB4* (NCBI accession: AGL08247) or *CpFatB5* (NCBI accession: AGL08248) under the control of the 1135 bp *B. napus Napin* promoter (NCBI accession: EU723261) were established using *B. nap*us “*Youngsan*”, as previously described [42]. *Youngsan* control plant and homozygous T4 transgenic plants were grown in greenhouse conditions located in the National Institute of Agricultural Sciences (Jeonju, Republic of Korea).

### 4.2. FA Analysis

Seed samples were heated at 90 °C for 90 min in 1 mL of 5% (*v*/*v*) H_2_SO_4_ in methanol and 0.3 mL of toluene with a known amount of 15:0 FAs as an internal standard. After the transmethylation, 1.5 mL of 0.9% NaCl solution was added, and FA methyl esters (FAMEs) were extracted with 1.5 mL of n-hexane three times. FAMEs were analyzed using gas chromatography with a 30 m × 0.25 mm (inner diameter) HP-FFAP column (Agilent, Pal Alto, CA, USA) with a GC-plus instrument (Shimadzu Corporation, Kyoto, Japan). The temperature program consisted of a 3 °C/min increase from 190 to 230 °C.

### 4.3. RNA Samples for RNA-Seq and Analysis of DEGs

Each flower bud was individually marked when it opened. Whole pods including developing seeds were harvested for RNA-Seq analyses at 8, 25, or 45 DAF. Nine RNA samples were prepared for the combination of three developmental stages (8, 25, or 45 DAF) and three genotypes (*Youngsan*, *CpFatB4*, or *CpFatB5* transgenic plants) (Figure 1a). Each sample was designated as follows: C1 (8 DAF), C2 (25 DAF), and C3 (45 DAF) for *Youngsan*; 41 (8 DAF), 42 (25 DAF), 43 (45 DAF) for *CpFatB4* transgenic plants; and 51 (8 DAF), 52 (25 DAF), and 53 (45 DAF) for *CpFatB5* transgenic plants. Each RNA sample for RNA-Seq was prepared by pooling equal amounts of three independently prepared total RNA to minimize the plant-by-plant variation in our analyses. Total RNA was isolated from one or two pods depending on the pod size using PureLink™ Plant RNA Reagent (Invitrogen, Carlsbad, CA, USA).

### 4.4. Illumina Sequencing, Data Processing, Reads Mapping, and Gene Annotation

cDNA libraries were constructed using an Illumina TruSeq RNA Sample Preparation Kit v2 (Illumina, San Diego, CA, USA) as instructed by the manufacturer, after the quality of each RNA sample was examined using a Bioanalyzer (Agilent, San Jose, CA, USA). The cDNA libraries were sequenced using Illumina HiSeq2000 with the paired-end approach. Raw RNA-seq datasets were deposited in the NCBI GEO (https://www.ncbi.nlm.nih.gov/geo/) under accession number GSE132071. Clean reads for DEG analysis were obtained from raw reads by trimming low-quality bases with Phred score (Q < 20) and removing the reads shorter than 25 bp using DynamicTrim and LengthSort in the SolexaQA package. Clean reads were mapped onto *B. napus* reference CDSs which were downloaded from Genoscope [8], using Bowtie2 (v2.1.0) software allowing up to 2 bp mismatches. The identified gene was annotated based on BLASTP results (e-value ≤ e^−10^) after CDS was translated into protein sequences.

### 4.5. DEG Analysis and Gene Annotation

The expression level of each gene was determined using read counts after the number of mapped reads for each gene was normalized using the DESeq package in R [43]. DEGs were selected based on the log2-fold change of gene expression (up-regulation for log2-fold change ≥ 1 and down-regulation for log2-fold change ≤ −1), and confirmed using the binomial test method with a false discovery rate (FDR) ≤ 0.05. Only the genes whose average expression levels were more than 1000 reads per gene were used to identify DEGs, except between samples C2 and 42, in which no DEGs were detected and a cutoff level of 200 reads per gene was used. C1_C2 indicates DEGs obtained from the comparison between C1 and C2 samples (Figure 1). Likewise, C2_C3, 41_42, 42_43, 51_52, and 52_53 are DEGs between different developmental stages, whereas C1_41, C1_51, C2_42, C2_52, C3_43, and C3_53 are DEGs between different genotypes at the same developmental stages. Annotation information was obtained from a custom BLASTP search against sequence information available at Phytozome and ARAPORT with the highest protein sequence similarity for each *B. napus* gene [25,26].

### 4.6. GO and KEGG Analysis

GO analysis was performed using the DEGs with normalized read number equal to or greater than 1000 that could be annotated with sequence information in ARAPORT and providing GO information. GO terms with five or more DEGs were identified for each comparison, among the molecular function, cellular component, and biological process categories. GO enrichment analyses for DEGs were performed using the Plant GeneSet Enrichment Analysis Toolkit, which supports analyses of *B. napus* [44]. *B. napus* genes were annotated using KEGG Orthology (KO) identifiers for KEGG analysis [29]. Up-regulated and down-regulated DEGs annotated with KO identifiers were mapped against the KEGG reference pathways using the Reconstruct Pathway in the KEGG mapper suite to identify the metabolic pathways affected by *CpFatB4* or *CpFatB5* overexpression. KEGG enrichment analyses for DEGs were also performed using the Plant GeneSet Enrichment Analysis Toolkit [44].

### 4.7. Real-Time Quantitative PCR

The first-strand cDNA synthesis reaction was performed using ReverTra Ace (Toyobo, Osaka, Japan) reverse transcriptase with oligo dT primer. RT-qPCR was performed on a CFX connect real-time PCR detection system (Bio-Rad, CA, USA) using SsoAdvanced Universal SYBR Green Supermix (Bio-Rad, CA, USA). The primer sequence information for *β-Actin*, *acyl-ACP thioesterase* (*FatA*), *palmitoyl-ACP thioesterase* (*FatB*), *Napin*,*β*-*ketoacyl-ACP synthase 2* (KASII), and *stearoyl-ACP desaturase* (*SAD*) genes were obtained from Hu et al. [45]. For *CpFatB4* detection, 5′-ATCCGCAAGGGTCTAACTCC-3′ and 5′-TCCACATTCCCGCCTGTATT-3′ were used. In the case of CpFatB5, 5′-ATATAGGCGGGAATGCGGAA-3′ and 5′-CAGTTCTGCCCTTCACGATG-3′ were used.

## 5. Conclusions

A deeper understanding of the regulation of lipid metabolism in developing seeds is essential for successful genetic engineering to improve oil yield or produce specialty oil in seeds. Using transgenic *B. napus*, we demonstrated that *CpFatB4* and *CpFatB5* originating from *C. paucipetala* had substrate preferences for palmitic and medium-chain acyl-ACP, respectively. Moreover, we uncovered gene expression changes responding to characteristic seed FA content changes in the transgenic plants. First, up-regulations of *KAS II* (elongation to 18:0 stearic acid) and desaturation genes (*stearoyl-ACP desaturase*, *oleate desaturase*, and *linoleate desaturase*) were observed in *CpFatB4* seeds with increased palmitic acid and decreased oleic acid contents. Second, continuous activations of storage protein genes such as *Napins* and *Cruciferins* were also observed in *CpFatB4* seeds. Third, altered expressions of many chloroplast-genome- and/or mitochondria-genome-originated genes were detected by *CpFatB5* overexpression in developing seeds. The gene expression changes in transgenic *B. napus* observed here may be induced to compensate the FA profile changes. Our findings will provide valuable information for further modifications towards acquiring desired oil compositions in *B. napus*.

## Figures and Tables

**Table 1:** Seed fatty acid profiles (mol %) and 100-seed weights showed different patterns depending on the transgene expressed. *Youngsan* (non-transgenic control plant, *n* = 12), *CpFatB4* (*n* = 10), and *CpFatB5* (*n* = 10). Seeds for each transgenic line were collected from T4 plants originating from the same T3 parent. Values are indicated as mean ± standard deviation.

Mol %	*Youngsan*	*CpFatB4*	*CpFatB5*
10:0	0.0	0.1	2.1 ± 0.3
12:0	0.0	0.1	1.0 ± 0.1
14:0	0.2	1.0 ± 0.1	0.6
16:0	5.3 ± 0.1	22.7 ± 1.7	9.0 ± 0.2
18:0	2.3 ± 0.1	3.4 ± 0.1	2.5 ± 0.1
18:1	67.8 ± 0.7	49.9±1.8	61.8 ± 0.7
18:2	16.6 ± 0.6	15.4 ± 0.7	15.7 ± 0.6
18:3	5.3 ± 0.4	4.7 ± 0.2	4.9 ± 0.2
20:0	0.8 ± 0.0	1.2 ± 0.0	0.8 ± 0.1
20:1	1.3 ± 0.0	1.0 ± 0.0	1.2 ± 0.0
22:1	0.3 ± 0.0	0.5 ± 0.0	0.4 ± 0.0
Percentage of saturated fatty acids	8.7	28.4	16.0
100-seed weight (mg)	227.4 ± 9.9	250.1 ± 9.3	216.1 ± 8.9

**Table 2:** Summary of DEG numbers in total transcriptome and lipid metabolism. In each comparison, the names of the two RNA samples compared are included in the comparison name, separated by an underscore. The percentages of lipid metabolism DEGs in the total DEGs are shown in the parentheses.

Type and Name of Comparisons		DEG Numbers		Up-Regulated DEG Numbers		Down-Regulated DEG Numbers	
		Total	Lipid Metabolism Genes (%)	Total	Lipid Metabolism Genes (%)	Total	Lipid Metabolism Genes (%)
Stage	C1_C2	2550	250 (9.8)	1688	159 (9.4)	862	91 (10.6)
	C2_C3	4642	267 (5.8)	2504	100 (4.0)	2138	167 (7.8)
	41_42	2486	244 (9.8)	1829	172 (9.4)	657	72 (11.0)
	42_43	2445	207 (8.5)	1028	72 (7.0)	1417	135 (9.5)
	51_52	2575	216 (8.4)	1692	141 (8.3)	883	75 (8.5)
	52_53	4813	264 (5.5)	1753	64 (3.7)	3060	200 (6.5)
Line	C1_41	366	10 (2.7)	174	0 (0)	192	10 (5.2)
	C2_42	45	0 (0)	24	0 (0)	21	0 (0)
	C3_43	1847	105 (5.7)	676	83 (12.3)	1171	22 (1.9)
	C1_51	289	6 (2.1)	155	0 (0)	134	6 (4.5)
	C2_52	112	6 (5.4)	12	1 (8.3)	100	5 (5.0)
	C3_53	1365	48 (3.5)	324	3 (0.9)	1041	45 (4.3)

## Supplementary Materials

Supplementary materials can be found at https://www.mdpi.com/1422-0067/20/13/3334/s1.

## Abbreviations

FA	Fatty acid
ACP	Acyl carrier protein
FatB	Fatty acyl-ACP thioesterase B
DAF	Days after flowering
