# Supplementary material for: Overexpression of Acyl-ACP Thioesterases, CpFatB4 and CpFatB5, Induce Distinct Gene Expression Reprogramming in Developing Seeds of Brassica napus
Source: Int J Mol Sci. 2019 Jul 6;20(13):3334. doi: 10.3390/ijms20133334 (PMC6651428; doi:10.3390/ijms20133334)
Supplement: Supplementary file 1 [file ijms-20-03334-s001.zip › TableS1.docx]

Table S1. Summary of Illumina sequencing data for nine samples used in RNA-seq analyses. Clean reads were mapped onto rapeseed reference CDS sequences.

| Sample name | Number of raw reads | Number of clean reads | Clean nucleotides (bp) | Number of genes expressed |
| --- | --- | --- | --- | --- |
| C1 | 34,376,946 | 32,180,622 | 2,958,923,472 | 81,007 |
| C2 | 37,825,752 | 35,405,452 | 3,250,870,352 | 81,383 |
| C3 | 35,471,510 | 32,772,978 | 2,968,889,309 | 78,914 |
| 41 | 35,369,166 | 33,065,832 | 3,023,153,116 | 81,512 |
| 42 | 35,394,894 | 32,980,978 | 3,008,523,534 | 80,766 |
| 43 | 31,994,608 | 29,154,724 | 2,608,286,439 | 76,094 |
| 51 | 32,513,910 | 29,367,418 | 2,650,833,406 | 80,836 |
| 52 | 37,665,202 | 34,408,734 | 3,076,974,543 | 82,033 |
| 53 | 30,346,586 | 27,517,552 | 2,449,904,434 | 76,454 |
| Total | 310,958,574 | 286,854,290 | 25,996,358,605 | 91,830 |
