# Supplementary material for: Overexpression of Acyl-ACP Thioesterases, CpFatB4 and CpFatB5, Induce Distinct Gene Expression Reprogramming in Developing Seeds of Brassica napus
Source: Int J Mol Sci. 2019 Jul 6;20(13):3334. doi: 10.3390/ijms20133334 (PMC6651428; doi:10.3390/ijms20133334)
Supplement: Supplementary file 1 [file ijms-20-03334-s001.zip › TableS10.docx]

Table S10. The top 20 lipid metabolism DEGs between growth stages or plant genotypes. Note that only 10 lipid metabolism DEGs were found in C1_41, and none in C2_42. UP and DOWN indicate expression changes of DEGs.

| Comparison |  | *B. napus* gene ID | TAIR gene ID | UP / DOWN | log2 fold change | Description |
| --- | --- | --- | --- | --- | --- | --- |
| C1_C2 | 1 | *BnaC03g65980D* | *AT4G34520* | UP | 12.49 | Ketoacyl-CoA Synthase |
|  | 2 | *BnaA02g11140D* | *AT3G56060* | UP | 12.42 | Glucose-methanol-choline (GMC) oxidoreductase family protein |
|  | 3 | *BnaC02g15450D* | *AT3G56060* | UP | 12.12 | Glucose-methanol-choline (GMC) oxidoreductase family protein |
|  | 4 | *BnaA08g11130D* | *AT4G34520* | UP | 12.11 | Ketoacyl-CoA Synthase |
|  | 5 | *BnaA07g14960D* | *AT5G40420* | UP | 11.89 | Oil-Body Oleosin |
|  | 6 | *BnaC06g12930D* | *AT5G40420* | UP | 11.88 | Oil-Body Oleosin |
|  | 7 | *BnaC07g39370D* | *AT4G25140* | UP | 11.67 | Oil-Body Oleosin |
|  | 8 | *BnaA08g14540D* | *AT4G25140* | UP | 11.58 | Oil-Body Oleosin |
|  | 9 | *BnaA03g47170D* | *AT4G25140* | UP | 11.49 | Oil-Body Oleosin |
|  | 10 | *BnaC01g17050D* | *AT4G25140* | UP | 11.49 | Oil-Body Oleosin |
|  | 11 | *BnaC08g11970D* | *AT4G25140* | UP | 11.47 | Oil-Body Oleosin |
|  | 12 | *BnaA01g14480D* | *AT4G25140* | UP | 11.38 | Oil-Body Oleosin |
|  | 13 | *BnaC04g32530D* | *AT5G40420* | UP | 11.24 | Oil-Body Oleosin |
|  | 14 | *BnaA05g33500D* | *AT3G02630* | UP | 10.57 | Stearoyl-ACP Desaturase |
|  | 15 | *BnaA03g37510D* | *AT3G24650* | UP | 10.50 | Homologous to the maize transcription factor Viviparous-1 |
|  | 16 | *BnaC05g48250D* | *AT3G02630* | UP | 10.49 | Stearoyl-ACP Desaturase |
|  | 17 | *BnaA09g02110D* | *AT3G27660* | UP | 9.48 | Oil-Body Oleosin |
|  | 18 | *BnaC02g37030D* | *AT3G27660* | UP | 9.25 | Oil-Body Oleosin |
|  | 19 | *BnaC08g12280D* | *AT4G26740* | UP | 9.23 | Caleosin |
|  | 20 | *BnaA01g15860D* | *AT4G26740* | UP | 9.08 | Caleosin |
| C2_C3 | 1 | *BnaA03g23490D* | *AT5G50600* | UP | 8.93 | Steroleosin |
|  | 2 | *BnaC03g27860D* | *AT5G50600* | UP | 8.91 | Steroleosin |
|  | 3 | *BnaA02g11570D* | *AT5G50600* | UP | 8.76 | Steroleosin |
|  | 4 | *BnaCnng57830D* | *AT5G50600* | UP | 8.75 | Steroleosin |
|  | 5 | *BnaA06g28680D* | *AT5G27600* | UP | 7.44 | Long-Chain Acyl-CoA Synthetase |
|  | 6 | *BnaC07g28060D* | *AT5G27600* | UP | 7.43 | Long-Chain Acyl-CoA Synthetase |
|  | 7 | *BnaA03g24220D* | *AT4G10020* | UP | 6.72 | Steroleosin |
|  | 8 | *BnaC03g28790D* | *AT4G10020* | UP | 6.70 | Steroleosin |
|  | 9 | *BnaA01g03200D* | *AT4G33790* | DOWN | -6.55 | Alcohol-forming Fatty Acyl-CoA Reductase |
|  | 10 | *BnaA05g33500D* | *AT3G02630* | DOWN | -6.50 | Stearoyl-ACP Desaturase |
|  | 11 | *BnaC01g04460D* | *AT4G33790* | DOWN | -6.50 | Alcohol-forming Fatty Acyl-CoA Reductase |
|  | 12 | *BnaC05g48250D* | *AT3G02630* | DOWN | -6.43 | Stearoyl-ACP Desaturase |
|  | 13 | *BnaA02g11140D* | *AT3G56060* | DOWN | -5.93 | Glucose-methanol-choline (GMC) oxidoreductase family protein |
|  | 14 | *BnaC02g15450D* | *AT3G56060* | DOWN | -5.91 | Glucose-methanol-choline (GMC) oxidoreductase family protein |
|  | 15 | *BnaC02g10060D* | *AT5G59310* | DOWN | -5.70 | Lipid Transfer Protein |
|  | 16 | *BnaA02g07120D* | *AT5G59310* | DOWN | -5.62 | Lipid Transfer Protein |
|  | 17 | *BnaA01g00820D* | *AT4G37060* | UP | 5.23 | Acyl-Hydrolase (Patatin-like) |
|  | 18 | *BnaC01g01830D* | *AT4G37060* | UP | 5.22 | Acyl-Hydrolase (Patatin-like) |
|  | 19 | *BnaA02g30470D* | *AT5G48880* | DOWN | -5.13 | Ketoacyl-CoA Thiolase |
|  | 20 | *BnaC02g38800D* | *AT5G48880* | DOWN | -5.12 | Ketoacyl-CoA Thiolase |
| 41_42 | 1 | *BnaA08g11130D* | *AT4G34520* | UP | 13.81 | Ketoacyl-CoA Synthase |
|  | 2 | *BnaC03g65980D* | *AT4G34520* | UP | 13.77 | Ketoacyl-CoA Synthase |
|  | 3 | *BnaA03g47170D* | *AT4G25140* | UP | 12.19 | Oil-Body Oleosin |
|  | 4 | *BnaA01g15860D* | *AT4G26740* | UP | 12.02 | Caleosin |
|  | 5 | *BnaC01g18950D* | *AT4G26740* | UP | 11.99 | Caleosin |
|  | 6 | *BnaC07g39370D* | *AT4G25140* | UP | 11.88 | Oil-Body Oleosin |
|  | 7 | *BnaC01g17050D* | *AT4G25140* | UP | 11.67 | Oil-Body Oleosin |
|  | 8 | *BnaC08g12280D* | *AT4G26740* | UP | 11.54 | Caleosin |
|  | 9 | *BnaA01g14480D* | *AT4G25140* | UP | 11.43 | Oil-Body Oleosin |
|  | 10 | *BnaA02g11140D* | *AT3G56060* | UP | 11.39 | Glucose-methanol-choline (GMC) oxidoreductase family protein |
|  | 11 | *BnaA08g14540D* | *AT4G25140* | UP | 11.37 | Oil-Body Oleosin |
|  | 12 | *BnaC08g11970D* | *AT4G25140* | UP | 11.37 | Oil-Body Oleosin |
|  | 13 | *BnaC02g15450D* | *AT3G56060* | UP | 11.19 | Glucose-methanol-choline (GMC) oxidoreductase family protein |
|  | 14 | *BnaC02g37030D* | *AT3G27660* | UP | 10.52 | Oil-Body Oleosin |
|  | 15 | *BnaA07g14960D* | *AT5G40420* | UP | 10.45 | Oil-Body Oleosin |
|  | 16 | *BnaC04g32530D* | *AT5G40420* | UP | 10.44 | Oil-Body Oleosin |
|  | 17 | *BnaC06g12930D* | *AT5G40420* | UP | 10.43 | Oil-Body Oleosin |
|  | 18 | *BnaA03g37510D* | *AT3G24650* | UP | 10.43 | Homologous to the maize transcription factor Viviparous-1 |
|  | 19 | *BnaA09g02110D* | *AT3G27660* | UP | 10.30 | Oil-Body Oleosin |
|  | 20 | *BnaC05g48250D* | *AT3G02630* | UP | 9.42 | Stearoyl-ACP Desaturase |
| 42_43 | 1 | *BnaC02g41520D* | *AT5G23940* | DOWN | -6.43 | DCR/PEL3 Acyltransferase |
|  | 2 | *BnaA03g23490D* | *AT5G50600* | UP | 6.06 | Steroleosin |
|  | 3 | *BnaC03g27860D* | *AT5G50600* | UP | 5.93 | Steroleosin |
|  | 4 | *BnaC02g10060D* | *AT5G59310* | DOWN | -5.91 | Lipid Transfer Protein |
|  | 5 | *BnaA02g07120D* | *AT5G59310* | DOWN | -5.79 | Lipid Transfer Protein |
|  | 6 | *BnaC01g04460D* | *AT4G33790* | DOWN | -5.78 | Alcohol-forming Fatty Acyl-CoA Reductase |
|  | 7 | *BnaCnng57830D* | *AT5G50600* | UP | 5.75 | Steroleosin |
|  | 8 | *BnaA01g03200D* | *AT4G33790* | DOWN | -5.74 | Alcohol-forming Fatty Acyl-CoA Reductase |
|  | 9 | *BnaA02g11570D* | *AT5G50600* | UP | 5.72 | Steroleosin |
|  | 10 | *BnaA02g07110D* | *AT5G59310* | DOWN | -5.31 | Lipid Transfer Protein |
|  | 11 | *BnaC04g06750D* | *AT2G38540* | DOWN | -5.27 | Lipid Transfer Protein |
|  | 12 | *BnaC02g10050D* | *AT5G59310* | DOWN | -5.21 | Lipid Transfer Protein |
|  | 13 | *BnaC03g74670D* | *AT5G63560* | UP | 5.20 | HXXXD-type acyl-transferase family protein |
|  | 14 | *BnaA06g22710D* | *AT5G63560* | UP | 5.11 | HXXXD-type acyl-transferase family protein |
|  | 15 | *BnaC01g01830D* | *AT4G37060* | UP | 5.10 | Acyl-Hydrolase (Patatin-like) |
|  | 16 | *BnaA01g00820D* | *AT4G37060* | UP | 5.09 | Acyl-Hydrolase (Patatin-like) |
|  | 17 | *BnaA10g13960D* | *ATCG00500* | UP | 4.97 | Carboxyltransferase beta Subunit of Heteromeric ACCase |
|  | 18 | *BnaC05g37990D* | *ATCG00500* | UP | 4.97 | Carboxyltransferase beta Subunit of Heteromeric ACCase |
|  | 19 | *BnaC09g27620D* | *ATCG00500* | UP | 4.97 | Carboxyltransferase beta Subunit of Heteromeric ACCase |
|  | 20 | *BnaC03g28790D* | *AT4G10020* | UP | 4.89 | Steroleosin |
| 51_52 | 1 | *BnaA02g11140D* | *AT3G56060* | UP | 11.12 | Glucose-methanol-choline (GMC) oxidoreductase family protein |
|  | 2 | *BnaC02g15450D* | *AT3G56060* | UP | 11.08 | Glucose-methanol-choline (GMC) oxidoreductase family protein |
|  | 3 | *BnaA08g11130D* | *AT4G34520* | UP | 11.08 | Ketoacyl-CoA Synthase |
|  | 4 | *BnaC03g65980D* | *AT4G34520* | UP | 11.04 | Ketoacyl-CoA Synthase |
|  | 5 | *BnaA03g37510D* | *AT3G24650* | UP | 10.16 | Homologous to the maize transcription factor Viviparous-1 |
|  | 6 | *BnaA05g33500D* | *AT3G02630* | UP | 10.08 | Stearoyl-ACP Desaturase |
|  | 7 | *BnaC01g17050D* | *AT4G25140* | UP | 10.02 | Oil-Body Oleosin |
|  | 8 | *BnaC05g48250D* | *AT3G02630* | UP | 9.99 | Stearoyl-ACP Desaturase |
|  | 9 | *BnaC08g11970D* | *AT4G25140* | UP | 9.96 | Oil-Body Oleosin |
|  | 10 | *BnaA08g14540D* | *AT4G25140* | UP | 9.87 | Oil-Body Oleosin |
|  | 11 | *BnaA07g14960D* | *AT5G40420* | UP | 9.79 | Oil-Body Oleosin |
|  | 12 | *BnaC06g12930D* | *AT5G40420* | UP | 9.79 | Oil-Body Oleosin |
|  | 13 | *BnaA01g14480D* | *AT4G25140* | UP | 9.74 | Oil-Body Oleosin |
|  | 14 | *BnaC07g39370D* | *AT4G25140* | UP | 9.60 | Oil-Body Oleosin |
|  | 15 | *BnaA03g47170D* | *AT4G25140* | UP | 9.50 | Oil-Body Oleosin |
|  | 16 | *BnaC04g32530D* | *AT5G40420* | UP | 9.37 | Oil-Body Oleosin |
|  | 17 | *BnaC02g37030D* | *AT3G27660* | UP | 7.96 | Oil-Body Oleosin |
|  | 18 | *BnaA02g29000D* | *AT3G27660* | UP | 7.88 | Oil-Body Oleosin |
|  | 19 | *BnaA09g02110D* | *AT3G27660* | UP | 7.45 | Oil-Body Oleosin |
|  | 20 | *BnaA04g15510D* | *AT2G26870* | UP | 5.55 | Phospholipase C (Non-specific) |
| 52_53 | 1 | *BnaCnng57830D* | *AT5G50600* | UP | 12.08 | Steroleosin |
|  | 2 | *BnaA02g11570D* | *AT5G50600* | UP | 12.01 | Steroleosin |
|  | 3 | *BnaC03g27860D* | *AT5G50600* | UP | 11.75 | Steroleosin |
|  | 4 | *BnaA03g23490D* | *AT5G50600* | UP | 11.55 | Steroleosin |
|  | 5 | *BnaA10g13960D* | *ATCG00500* | UP | 8.52 | Carboxyltransferase beta Subunit of Heteromeric ACCase |
|  | 6 | *BnaC05g37990D* | *ATCG00500* | UP | 8.52 | Carboxyltransferase beta Subunit of Heteromeric ACCase |
|  | 7 | *BnaC09g27620D* | *ATCG00500* | UP | 8.52 | Carboxyltransferase beta Subunit of Heteromeric ACCase |
|  | 8 | *BnaC03g28790D* | *AT4G10020* | UP | 7.89 | Steroleosin |
|  | 9 | *BnaA03g24220D* | *AT4G10020* | UP | 7.87 | Steroleosin |
|  | 10 | *BnaA01g00820D* | *AT4G37060* | UP | 7.86 | Acyl-Hydrolase (Patatin-like) |
|  | 11 | *BnaC01g01830D* | *AT4G37060* | UP | 7.85 | Acyl-Hydrolase (Patatin-like) |
|  | 12 | *BnaA02g11140D* | *AT3G56060* | DOWN | -7.80 | Glucose-methanol-choline (GMC) oxidoreductase family protein |
|  | 13 | *BnaA01g03200D* | *AT4G33790* | DOWN | -7.70 | Alcohol-forming Fatty Acyl-CoA Reductase |
|  | 14 | *BnaC02g15450D* | *AT3G56060* | DOWN | -7.68 | Glucose-methanol-choline (GMC) oxidoreductase family protein |
|  | 15 | *BnaA05g33500D* | *AT3G02630* | DOWN | -7.56 | Stearoyl-ACP Desaturase |
|  | 16 | *BnaC01g04460D* | *AT4G33790* | DOWN | -7.48 | Alcohol-forming Fatty Acyl-CoA Reductase |
|  | 17 | *BnaC05g48250D* | *AT3G02630* | DOWN | -7.47 | Stearoyl-ACP Desaturase |
|  | 18 | *BnaC07g28060D* | *AT5G27600* | UP | 7.46 | Long-Chain Acyl-CoA Synthetase |
|  | 19 | *BnaA06g28680D* | *AT5G27600* | UP | 7.45 | Long-Chain Acyl-CoA Synthetase |
|  | 20 | *BnaC02g10060D* | *AT5G59310* | DOWN | -7.07 | Lipid Transfer Protein |
| C1_41 | 1 | *BnaC04g41130D* | *AT2G30490* | DOWN | -1.16 | Cinnamate 4-Hydroxylase |
|  | 2 | *BnaC03g16960D* | *AT2G30490* | DOWN | -1.15 | Cinnamate 4-Hydroxylase |
|  | 3 | *BnaA04g17570D* | *AT2G30490* | DOWN | -1.14 | Cinnamate 4-Hydroxylase |
|  | 4 | *BnaA05g11950D* | *AT2G30490* | DOWN | -1.12 | Cinnamate 4-Hydroxylase |
|  | 5 | *BnaA01g34710D* | *AT4G39800* | DOWN | -1.12 | myo-inositol-3-phosphate synthase |
|  | 6 | *BnaC04g14330D* | *AT2G30490* | DOWN | -1.12 | Cinnamate 4-Hydroxylase |
|  | 7 | *BnaC01g00680D* | *AT4G39800* | DOWN | -1.11 | myo-inositol-3-phosphate synthase |
|  | 8 | *BnaA03g14010D* | *AT2G30490* | DOWN | -1.11 | Cinnamate 4-Hydroxylase |
|  | 9 | *BnaA09g42760D* | *AT2G22240* | DOWN | -1.04 | myo-inositol-3-phosphate synthase |
|  | 10 | *BnaC08g35240D* | *AT2G22240* | DOWN | -1.01 | myo-inositol-3-phosphate synthase |
| C3_43 | 1 | *BnaC09g54530D* | *AT5G14180* | DOWN | -2.97 | Triacylglycerol Lipase (TAGL) |
|  | 2 | *BnaA10g19480D* | *AT5G14180* | DOWN | -2.93 | Triacylglycerol Lipase (TAGL) |
|  | 3 | *BnaA04g17150D* | *AT2G29980* | UP | 2.79 | Linoleate Desaturase |
|  | 4 | *BnaA10g13960D* | *ATCG00500* | UP | 2.77 | Carboxyltransferase beta Subunit of Heteromeric ACCase |
|  | 5 | *BnaC05g37990D* | *ATCG00500* | UP | 2.77 | Carboxyltransferase beta Subunit of Heteromeric ACCase |
|  | 6 | *BnaC09g27620D* | *ATCG00500* | UP | 2.77 | Carboxyltransferase beta Subunit of Heteromeric ACCase |
|  | 7 | *BnaC04g40760D* | *AT2G29980* | UP | 2.77 | Linoleate Desaturase |
|  | 8 | *BnaA03g13590D* | *AT2G29980* | UP | 2.65 | Linoleate Desaturase |
|  | 9 | *BnaC03g16520D* | *AT2G29980* | UP | 2.62 | Linoleate Desaturase |
|  | 10 | *BnaA08g11130D* | *AT4G34520* | UP | 2.55 | Ketoacyl-CoA Synthase |
|  | 11 | *BnaC03g65980D* | *AT4G34520* | UP | 2.54 | Ketoacyl-CoA Synthase |
|  | 12 | *BnaC04g14820D* | *AT2G29980* | UP | 2.54 | Linoleate Desaturase |
|  | 13 | *BnaC03g74670D* | *AT5G63560* | UP | 2.53 | HXXXD-type acyl-transferase family protein |
|  | 14 | *BnaA06g22710D* | *AT5G63560* | UP | 2.52 | HXXXD-type acyl-transferase family protein |
|  | 15 | *BnaA03g20420D* | *AT2G43710* | UP | 2.50 | Stearoyl-ACP Desaturase |
|  | 16 | *BnaA05g12360D* | *AT2G29980* | UP | 2.48 | Linoleate Desaturase |
|  | 17 | *BnaC03g24420D* | *AT2G43710* | UP | 2.43 | Stearoyl-ACP Desaturase |
|  | 18 | *BnaA10g18080D* | *AT5G16240* | UP | 2.38 | Stearoyl-ACP Desaturase |
|  | 19 | *BnaC09g41580D* | *AT5G16240* | UP | 2.30 | Stearoyl-ACP Desaturase |
|  | 20 | *BnaC05g49350D* | *AT1G13640* | DOWN | -2.25 | Phosphatidylinositol-4-Kinase gamma |
| C1_51 | 1 | *BnaC01g00680D* | *AT4G39800* | DOWN | -1.38 | myo-inositol-3-phosphate synthase |
|  | 2 | *BnaA01g34710D* | *AT4G39800* | DOWN | -1.34 | myo-inositol-3-phosphate synthase |
|  | 3 | *BnaA09g42760D* | *AT2G22240* | DOWN | -1.31 | myo-inositol-3-phosphate synthase |
|  | 4 | *BnaC08g35240D* | *AT2G22240* | DOWN | -1.31 | myo-inositol-3-phosphate synthase |
|  | 5 | *BnaC04g34650D* | *AT2G22240* | DOWN | -1.23 | myo-inositol-3-phosphate synthase |
|  | 6 | *BnaA04g12720D* | *AT2G22240* | DOWN | -1.23 | myo-inositol-3-phosphate synthase |
| C2_52 | 1 | *BnaC01g18950D* | *AT4G26740* | DOWN | -1.20 | Caleosin |
|  | 2 | *BnaA01g15860D* | *AT4G26740* | DOWN | -1.19 | Caleosin |
|  | 3 | *BnaC08g12280D* | *AT4G26740* | DOWN | -1.19 | Caleosin |
|  | 4 | *BnaA09g02110D* | *AT3G27660* | DOWN | -1.10 | Oil-Body Oleosin |
|  | 5 | *BnaCnng38620D* | *AT3G45140* | UP | 1.08 | Lipoxygenase |
|  | 6 | *BnaA02g29000D* | *AT3G27660* | DOWN | -1.07 | Oil-Body Oleosin |
| C3_53 | 1 | *BnaA10g13960D* | *ATCG00500* | UP | 4.10 | Carboxyltransferase beta Subunit of Heteromeric ACCase |
|  | 2 | *BnaC05g37990D* | *ATCG00500* | UP | 4.10 | Carboxyltransferase beta Subunit of Heteromeric ACCase |
|  | 3 | *BnaC09g27620D* | *ATCG00500* | UP | 4.10 | Carboxyltransferase beta Subunit of Heteromeric ACCase |
|  | 4 | *BnaA07g24880D* | *AT3G45140* | DOWN | -2.61 | Lipoxygenase |
|  | 5 | *BnaA07g38540D* | *AT3G45140* | DOWN | -2.46 | Lipoxygenase |
|  | 6 | *BnaA07g38550D* | *AT3G45140* | DOWN | -2.43 | Lipoxygenase |
|  | 7 | *BnaA02g11430D* | *AT3G45140* | DOWN | -2.41 | Lipoxygenase |
|  | 8 | *BnaC02g15950D* | *AT3G45140* | DOWN | -2.34 | Lipoxygenase |
|  | 9 | *BnaA07g24860D* | *AT3G45140* | DOWN | -2.34 | Lipoxygenase |
|  | 10 | *BnaC06g26190D* | *AT3G45140* | DOWN | -2.33 | Lipoxygenase |
|  | 11 | *BnaC06g18870D* | *AT3G45140* | DOWN | -2.08 | Lipoxygenase |
|  | 12 | *BnaA07g19600D* | *AT3G45140* | DOWN | -2.06 | Lipoxygenase |
|  | 13 | *BnaA08g11130D* | *AT4G34520* | DOWN | -1.57 | Ketoacyl-CoA Synthase |
|  | 14 | *BnaC03g65980D* | *AT4G34520* | DOWN | -1.56 | Ketoacyl-CoA Synthase |
|  | 15 | *BnaC06g01550D* | *AT1G48030* | DOWN | -1.50 | Mitochondrial Dihydrolipoamide Dehydrogenase, branched chain alpha-ketoacid dehydrogenase complex |
|  | 16 | *BnaA06g04090D* | *AT1G48030* | DOWN | -1.47 | Mitochondrial Dihydrolipoamide Dehydrogenase, branched chain alpha-ketoacid dehydrogenase complex |
|  | 17 | *BnaC01g35830D* | *AT3G15730* | DOWN | -1.47 | Phospholipase D alpha |
|  | 18 | *BnaC08g46110D* | *AT1G01090* | DOWN | -1.39 | Pyruvate Dehydrogenase alpha subunit, E1a component of Pyruvate Dehydrogenase Complex |
|  | 19 | *BnaA05g20960D* | *AT3G19240* | DOWN | -1.39 | Phosphoinositide 3-Phosphatase |
|  | 20 | *BnaA01g28530D* | *AT3G15730* | DOWN | -1.39 | Phospholipase D alpha |
