# Supplementary material for: Overexpression of Acyl-ACP Thioesterases, CpFatB4 and CpFatB5, Induce Distinct Gene Expression Reprogramming in Developing Seeds of Brassica napus
Source: Int J Mol Sci. 2019 Jul 6;20(13):3334. doi: 10.3390/ijms20133334 (PMC6651428; doi:10.3390/ijms20133334)
Supplement: Supplementary file 1 [file ijms-20-03334-s001.zip › TableS5.docx]

**Table S5**. Top 20 genes showing the strongest differential expression between developmental stages in 3 genotypes. C1_C2 indicates DEGs obtained from comparison between C1 and C2 samples. Likewise, C2_C3, 41_42, 42_43, 51_52, 52_53 are DEGs between different developmental stages in a genotype.

| Comparison |  | *Reference CDS (Brassica napus)* | *TAIR Gene ID* | Up or down | log2 fold change | Description |
| --- | --- | --- | --- | --- | --- | --- |
| C1_C2 | 1 | *BnaA08g15380D* | *AT4G36700* | up | 14.11 | RmlC-like cupins superfamily protein |
|  | 2 | *BnaC07g18750D* | *AT5G45890* | up | 13.53 | senescence-associated gene 12 |
|  | 3 | *BnaC03g61870D* | *AT4G36700* | up | 13.28 | RmlC-like cupins superfamily protein |
|  | 4 | *BnaA06g40240D* | *AT5G45890* | up | 13.23 | senescence-associated gene 12 |
|  | 5 | *BnaC07g46180D* | *AT4G36700* | up | 13.23 | RmlC-like cupins superfamily protein |
|  | 6 | *BnaC02g38340D* | *AT5G48100* | up | 12.86 | Laccase/Diphenol oxidase family protein |
|  | 7 | *BnaC06g01870D* | *AT4G37900* | up | 12.81 | Protein of unknown function (duplicated DUF1399) |
|  | 8 | *BnaC03g65980D* | *AT4G34520* | up | 12.49 | 3-ketoacyl-CoA synthase 18 |
|  | 9 | *BnaA02g11140D* | *AT3G56060* | up | 12.42 | Glucose-methanol-choline (GMC) oxidoreductase family protein |
|  | 10 | *BnaAnng08030D* | *AT5G48100* | up | 12.39 | Laccase/Diphenol oxidase family protein |
|  | 11 | *BnaA06g03920D* | *AT4G37900* | up | 12.35 | Protein of unknown function (duplicated DUF1399) |
|  | 12 | *BnaC04g39100D* | *AT3G22142* | up | 12.34 | Bifunctional inhibitor/lipid-transfer protein/seed storage 2S albumin superfamily protein |
|  | 13 | *BnaC09g48350D* | *AT5G07190* | up | 12.34 | seed gene 3 |
|  | 14 | *BnaAnng25140D* | *AT4G37900* | up | 12.14 | Protein of unknown function (duplicated DUF1399) |
|  | 15 | *BnaC02g15450D* | *AT3G56060* | up | 12.12 | Glucose-methanol-choline (GMC) oxidoreductase family protein |
|  | 16 | *BnaA08g11130D* | *AT4G34520* | up | 12.11 | 3-ketoacyl-CoA synthase 18 |
|  | 17 | *BnaC05g02160D* | *AT1G03880* | up | 12.06 | cruciferin 2 |
|  | 18 | *BnaA10g02240D* | *AT1G03880* | up | 12.05 | cruciferin 2 |
|  | 19 | *BnaC04g30640D* | *AT5G38195* | up | 11.92 | Bifunctional inhibitor/lipid-transfer protein/seed storage 2S albumin superfamily protein |
|  | 20 | *BnaA10g23630D* | *AT5G07190* | up | 11.92 | seed gene 3 |
| C2_C3 | 1 | *BnaA05g02110D* | *AT2G41280* | up | 14.41 | late embryogenesis abundant protein (LEA) protein M10 |
|  | 2 | *BnaC03g23240D* | *AT2G41280* | up | 14.38 | late embryogenesis abundant protein (LEA) protein M10 |
|  | 3 | *BnaC05g11200D* | *AT3G21380* | up | 14.18 | Mannose-binding lectin superfamily protein |
|  | 4 | *BnaA04g24580D* | *AT2G42560* | up | 14.09 | late embryogenesis abundant domain-containing protein / LEA domain-containing protein |
|  | 5 | *BnaC04g48420D* | *AT2G42560* | up | 14.09 | late embryogenesis abundant domain-containing protein / LEA domain-containing protein |
|  | 6 | *BnaA06g09710D* | *AT3G21380* | up | 14.02 | Mannose-binding lectin superfamily protein |
|  | 7 | *BnaC04g01780D* | *AT2G41280* | up | 13.62 | late embryogenesis abundant protein (LEA) protein M10 |
|  | 8 | *BnaC04g47570D* | *AT2G41280* | up | 13.44 | late embryogenesis abundant protein (LEA) protein M10 |
|  | 9 | *BnaA04g23790D* | *AT2G41280* | up | 13.40 | late embryogenesis abundant protein (LEA) protein M10 |
|  | 10 | *BnaA03g18930D* | *AT2G40170* | up | 13.32 | Stress induced protein |
|  | 11 | *BnaC03g22490D* | *AT2G40170* | up | 13.15 | Stress induced protein |
|  | 12 | *BnaA01g24560D* | *AT3G22640* | up | 13.06 | cupin family protein |
|  | 13 | *BnaCnng28630D* | *AT3G22640* | up | 12.93 | cupin family protein |
|  | 14 | *BnaC01g43710D* | *AT3G21380* | up | 12.91 | Mannose-binding lectin superfamily protein |
|  | 15 | *BnaA05g17150D* | *AT3G22490* | up | 12.59 | Seed maturation protein |
|  | 16 | *BnaC05g29980D* | *AT3G22490* | up | 12.59 | Seed maturation protein |
|  | 17 | *BnaC05g33270D* | *AT3G21380* | up | 12.56 | Mannose-binding lectin superfamily protein |
|  | 18 | *BnaA09g18220D* | *AT2G05580* | up | 12.54 | Glycine-rich protein family |
|  | 19 | *BnaC03g19110D* | *AT2G34740* | up | 12.53 | Protein phosphatase 2C family protein |
|  | 20 | *BnaC05g29930D* | *AT3G22490* | up | 12.45 | Seed maturation protein |
| 41_42 | 1 | *BnaC07g40770D* | *AT4G27160* | up | 15.28 | seed storage albumin 3 |
|  | 2 | *BnaA03g48490D* | *AT4G27160* | up | 15.19 | seed storage albumin 3 |
|  | 3 | *BnaC07g48660D* | *AT5G44120* | up | 14.66 | RmlC-like cupins superfamily protein |
|  | 4 | *BnaA06g36310D* | *AT5G44120* | up | 14.51 | RmlC-like cupins superfamily protein |
|  | 5 | *BnaA01g08350D* | *AT4G28520* | up | 14.35 | cruciferin 3 |
|  | 6 | *BnaC01g09900D* | *AT4G28520* | up | 14.29 | cruciferin 3 |
|  | 7 | *BnaA08g13680D* | *AT5G44120* | up | 14.27 | RmlC-like cupins superfamily protein |
|  | 8 | *BnaA02g22500D* | *AT5G44120* | up | 14.06 | RmlC-like cupins superfamily protein |
|  | 9 | *BnaA07g13950D* | *AT2G28490* | up | 13.82 | RmlC-like cupins superfamily protein |
|  | 10 | *BnaA08g11130D* | *AT4G34520* | up | 13.81 | 3-ketoacyl-CoA synthase 18 |
|  | 11 | *BnaCnng58970D* | *AT5G44120* | up | 13.79 | RmlC-like cupins superfamily protein |
|  | 12 | *BnaC03g65980D* | *AT4G34520* | up | 13.77 | 3-ketoacyl-CoA synthase 18 |
|  | 13 | *BnaA10g02240D* | *AT1G03880* | up | 13.30 | cruciferin 2 |
|  | 14 | *BnaC05g02160D* | *AT1G03880* | up | 13.27 | cruciferin 2 |
|  | 15 | *BnaC03g61870D* | *AT4G36700* | up | 12.88 | RmlC-like cupins superfamily protein |
|  | 16 | *BnaA08g15380D* | *AT4G36700* | up | 12.88 | RmlC-like cupins superfamily protein |
|  | 17 | *BnaA04g15830D* | *AT3G22142* | up | 12.67 | Bifunctional inhibitor/lipid-transfer protein/seed storage 2S albumin superfamily protein |
|  | 18 | *BnaC04g30640D* | *AT5G38195* | up | 12.65 | Bifunctional inhibitor/lipid-transfer protein/seed storage 2S albumin superfamily protein |
|  | 19 | *BnaC02g38340D* | *AT5G48100* | up | 12.41 | Laccase/Diphenol oxidase family protein |
|  | 20 | *BnaAnng08030D* | *AT5G48100* | up | 12.37 | Laccase/Diphenol oxidase family protein |
| 42_43 | 1 | *BnaA05g02110D* | *AT2G41280* | up | 11.58 | late embryogenesis abundant protein (LEA) protein M10 |
|  | 2 | *BnaA03g38820D* | *AT4G23690* | down | -11.28 | Disease resistance-responsive (dirigent-like protein) family protein |
|  | 3 | *BnaC03g45710D* | *AT4G23690* | down | -11.28 | Disease resistance-responsive (dirigent-like protein) family protein |
|  | 4 | *BnaC04g47570D* | *AT2G41280* | up | 10.99 | late embryogenesis abundant protein (LEA) protein M10 |
|  | 5 | *BnaA04g23790D* | *AT2G41280* | up | 10.96 | late embryogenesis abundant protein (LEA) protein M10 |
|  | 6 | *BnaC05g29980D* | *AT3G22490* | up | 10.46 | Seed maturation protein |
|  | 7 | *BnaA05g17150D* | *AT3G22490* | up | 10.45 | Seed maturation protein |
|  | 8 | *BnaC05g29930D* | *AT3G22490* | up | 10.32 | Seed maturation protein |
|  | 9 | *BnaC04g01780D* | *AT2G41280* | up | 10.14 | late embryogenesis abundant protein (LEA) protein M10 |
|  | 10 | *BnaC03g23240D* | *AT2G41280* | up | 10.12 | late embryogenesis abundant protein (LEA) protein M10 |
|  | 11 | *BnaA05g08680D* | *AT2G35300* | up | 9.68 | Late embryogenesis abundant protein, group 1 protein |
|  | 12 | *BnaC04g09820D* | *AT2G35300* | up | 9.55 | Late embryogenesis abundant protein, group 1 protein |
|  | 13 | *BnaA06g09710D* | *AT3G21380* | up | 9.50 | Mannose-binding lectin superfamily protein |
|  | 14 | *BnaC03g22490D* | *AT2G40170* | up | 9.45 | Stress induced protein |
|  | 15 | *BnaC05g11200D* | *AT3G21380* | up | 9.38 | Mannose-binding lectin superfamily protein |
|  | 16 | *BnaC05g02070D* | *AT1G03790* | up | 9.25 | Zinc finger C-x8-C-x5-C-x3-H type family protein |
|  | 17 | *BnaA10g02150D* | *AT1G03790* | up | 9.23 | Zinc finger C-x8-C-x5-C-x3-H type family protein |
|  | 18 | *BnaC01g43710D* | *AT3G21380* | up | 9.13 | Mannose-binding lectin superfamily protein |
|  | 19 | *BnaC02g31840D* | *AT4G32110* | down | -9.03 | Beta-1,3-N-Acetylglucosaminyltransferase family protein |
|  | 20 | *BnaA02g24080D* | *AT4G32110* | down | -8.94 | Beta-1,3-N-Acetylglucosaminyltransferase family protein |
| 51_52 | 1 | *BnaA06g03920D* | *AT4G37900* | up | 13.59 | Protein of unknown function (duplicated DUF1399) |
|  | 2 | *BnaC06g01870D* | *AT4G37900* | up | 13.18 | Protein of unknown function (duplicated DUF1399) |
|  | 3 | *BnaC05g43050D* | *AT3G09950* | up | 12.98 | - |
|  | 4 | *BnaAnng25140D* | *AT4G37900* | up | 12.61 | Protein of unknown function (duplicated DUF1399) |
|  | 5 | *BnaA06g40240D* | *AT5G45890* | up | 12.47 | senescence-associated gene 12 |
|  | 6 | *BnaC04g30640D* | *AT5G38195* | up | 12.38 | Bifunctional inhibitor/lipid-transfer protein/seed storage 2S albumin superfamily protein |
|  | 7 | *BnaC07g18750D* | *AT5G45890* | up | 12.14 | senescence-associated gene 12 |
|  | 8 | *BnaC02g38340D* | *AT5G48100* | up | 11.96 | Laccase/Diphenol oxidase family protein |
|  | 9 | *BnaAnng08030D* | *AT5G48100* | up | 11.92 | Laccase/Diphenol oxidase family protein |
|  | 10 | *BnaC08g43960D* | *AT1G04660* | up | 11.82 | glycine-rich protein |
|  | 11 | *BnaC02g18030D* | *AT5G05020* | up | 11.81 | Pollen Ole e 1 allergen and extensin family protein |
|  | 12 | *BnaAnng12280D* | *AT1G04660* | up | 11.81 | glycine-rich protein |
|  | 13 | *BnaA02g13680D* | *AT5G05020* | up | 11.81 | Pollen Ole e 1 allergen and extensin family protein |
|  | 14 | *BnaC09g48350D* | *AT5G07190* | up | 11.56 | seed gene 3 |
|  | 15 | *BnaA03g32070D* | *AT4G21380* | down | -11.43 | receptor kinase 3 |
|  | 16 | *BnaC03g37350D* | *AT4G21380* | down | -11.41 | receptor kinase 3 |
|  | 17 | *BnaC04g39100D* | *AT3G22142* | up | 11.36 | Bifunctional inhibitor/lipid-transfer protein/seed storage 2S albumin superfamily protein |
|  | 18 | *BnaA08g15380D* | *AT4G36700* | up | 11.21 | RmlC-like cupins superfamily protein |
|  | 19 | *BnaC03g61870D* | *AT4G36700* | up | 11.16 | RmlC-like cupins superfamily protein |
|  | 20 | *BnaA10g23630D* | *AT5G07190* | up | 11.12 | seed gene 3 |
| 52_53 | 1 | *BnaA04g23790D* | *AT2G41280* | up | 14.85 | late embryogenesis abundant protein (LEA) protein M10 |
|  | 2 | *BnaA01g24560D* | *AT3G22640* | up | 14.84 | cupin family protein |
|  | 3 | *BnaC04g47570D* | *AT2G41280* | up | 14.70 | late embryogenesis abundant protein (LEA) protein M10 |
|  | 4 | *BnaCnng28630D* | *AT3G22640* | up | 14.59 | cupin family protein |
|  | 5 | *BnaC03g23240D* | *AT2G41280* | up | 14.02 | late embryogenesis abundant protein (LEA) protein M10 |
|  | 6 | *BnaC05g11200D* | *AT3G21380* | up | 13.91 | Mannose-binding lectin superfamily protein |
|  | 7 | *BnaA05g34530D* | *AT2G40170* | up | 13.90 | Stress induced protein |
|  | 8 | *BnaCnng27950D* | *AT2G40170* | up | 13.89 | Stress induced protein |
|  | 9 | *BnaA06g09710D* | *AT3G21380* | up | 13.56 | Mannose-binding lectin superfamily protein |
|  | 10 | *BnaCnng07050D* | *AT3G21720* | up | 13.41 | isocitrate lyase |
|  | 11 | *BnaA05g17150D* | *AT3G22490* | up | 13.28 | Seed maturation protein |
|  | 12 | *BnaC05g29980D* | *AT3G22490* | up | 13.28 | Seed maturation protein |
|  | 13 | *BnaC05g29930D* | *AT3G22490* | up | 13.28 | Seed maturation protein |
|  | 14 | *BnaA09g18220D* | *AT2G05580* | up | 13.20 | Glycine-rich protein family |
|  | 15 | *BnaA05g19530D* | *AT3G21720* | up | 13.18 | isocitrate lyase |
|  | 16 | *BnaA05g02110D* | *AT2G41280* | up | 13.17 | late embryogenesis abundant protein (LEA) protein M10 |
|  | 17 | *BnaA04g24580D* | *AT2G42560* | up | 13.14 | late embryogenesis abundant domain-containing protein / LEA domain-containing protein |
|  | 18 | *BnaA03g36420D* | *AT3G21720* | up | 13.04 | isocitrate lyase |
|  | 19 | *BnaC03g42310D* | *AT3G21720* | up | 13.04 | isocitrate lyase |
|  | 20 | *BnaC05g33270D* | *AT3G21380* | up | 12.90 | Mannose-binding lectin superfamily protein |
