# Supplementary material for: Overexpression of Acyl-ACP Thioesterases, CpFatB4 and CpFatB5, Induce Distinct Gene Expression Reprogramming in Developing Seeds of Brassica napus
Source: Int J Mol Sci. 2019 Jul 6;20(13):3334. doi: 10.3390/ijms20133334 (PMC6651428; doi:10.3390/ijms20133334)
Supplement: Supplementary file 1 [file ijms-20-03334-s001.zip › TableS6.docx]

**Table S6**. Top 20 genes showing the strongest differential expression between genotypes at a specific developmental stage. C1_41 indicates DEGs obtained from comparison between C1 and 41 samples. Likewise, C2_42, C3_43, C1_51, C2_52, C3_53 are DEGs between different lines at the same developmental stage. * Rice gene ID; ** Rice description

| Comparison |  | Reference CDS (*Brassica napus*) | TAIR Gene ID | Up or down | log2 fold change | Description |
| --- | --- | --- | --- | --- | --- | --- |
| C1_41 | 1 | *BnaC08g4936D* | *AT2G21660* | up | 3.27 | cold, circadian rhythm, and RNA binding 2 |
|  | 2 | *BnaUnng05060D* | *AT2G21660* | up | 3.13 | cold, circadian rhythm, and RNA binding 2 |
|  | 3 | *BnaA04g12350D* | *AT2G21660* | up | 3.08 | cold, circadian rhythm, and RNA binding 2 |
|  | 4 | *BnaAnng35580D* | *AT2G21660* | up | 3.03 | cold, circadian rhythm, and RNA binding 2 |
|  | 5 | *BnaC05g00840D* | *AT1G01060* | down | -2.76 | Homeodomain-like superfamily protein |
|  | 6 | *BnaA10g00780D* | *AT1G01060* | down | -2.76 | Homeodomain-like superfamily protein |
|  | 7 | *BnaA06g04960D* | *AT1G08570* | down | -2.69 | atypical CYS HIS rich thioredoxin 4 |
|  | 8 | *BnaC05g06220D* | *AT1G08570* | down | -2.61 | atypical CYS HIS rich thioredoxin 4 |
|  | 9 | *BnaA08g16610D* | *AT4G39260* | up | 2.60 | cold, circadian rhythm, and RNA binding 1 |
|  | 10 | *BnaC03g37520D* | *AT3G12320* | down | -2.59 | - |
|  | 11 | *BnaA03g32230D* | *AT3G12320* | down | -2.55 | - |
|  | 12 | *BnaC03g60490D* | *AT4G39260* | up | 2.45 | cold, circadian rhythm, and RNA binding 1 |
|  | 13 | *BnaA04g23610D* | *AT2G40900* | down | -2.41 | nodulin MtN21 /EamA-like transporter family protein |
|  | 14 | *BnaA01g05410D* | *AT4G39260* | up | 2.39 | cold, circadian rhythm, and RNA binding 1 |
|  | 15 | *BnaC04g47490D* | *AT2G40900* | down | -2.37 | nodulin MtN21 /EamA-like transporter family protein |
|  | 16 | *BnaCnng57410D* | *AT2G40900* | down | -2.36 | nodulin MtN21 /EamA-like transporter family protein |
|  | 17 | *BnaA09g48840D* | *AT1G08570* | down | -2.31 | atypical CYS HIS rich thioredoxin 4 |
|  | 18 | *BnaC01g00280D* | *AT4G39260* | up | 2.26 | cold, circadian rhythm, and RNA binding 1 |
|  | 19 | *BnaC09g16520D* | *LOC_Os05g22840.1** | up | 2.26 | expressed protein** |
|  | 20 | *BnaA04g06320D* | *LOC_Os05g22840.1** | up | 2.26 | expressed protein** |
| C2_42 | 1 | *BnaA07g35640D* | *AT1G80440* | up | 2.61 | Galactose oxidase/kelch repeat superfamily protein |
|  | 2 | *BnaCnng58970D* | *AT5G44120* | up | 1.77 | RmlC-like cupins superfamily protein |
|  | 3 | *BnaC06g14690D* | *ATCG00020* | down | -1.77 | photosystem II reaction center protein A |
|  | 4 | *BnaC03g33010D* | *AT3G02550* | up | 1.61 | LOB domain-containing protein 41 |
|  | 5 | *BnaA08g13680D* | *AT5G44120* | up | 1.60 | RmlC-like cupins superfamily protein |
|  | 6 | *BnaA06g36310D* | *AT5G44120* | up | 1.60 | RmlC-like cupins superfamily protein |
|  | 7 | *BnaA03g27980D* | *AT3G02550* | up | 1.59 | LOB domain-containing protein 41 |
|  | 8 | *BnaC07g48660D* | *AT5G44120* | up | 1.58 | RmlC-like cupins superfamily protein |
|  | 9 | *BnaA02g22500D* | *AT5G44120* | up | 1.58 | RmlC-like cupins superfamily protein |
|  | 10 | *BnaA01g08350D* | *AT4G28520* | up | 1.54 | cruciferin 3 |
|  | 11 | *BnaC01g09900D* | *AT4G28520* | up | 1.53 | cruciferin 3 |
|  | 12 | *BnaA07g37260D* | *AT5G07470* | up | 1.32 | peptidemethionine sulfoxide reductase 3 |
|  | 13 | *BnaA03g02060D* | *AT5G07470* | up | 1.31 | peptidemethionine sulfoxide reductase 3 |
|  | 14 | *BnaC03g03070D* | *AT5G07470* | up | 1.31 | peptidemethionine sulfoxide reductase 3 |
|  | 15 | *BnaC03g03090D* | *AT5G07470* | up | 1.31 | peptidemethionine sulfoxide reductase 3 |
|  | 16 | *BnaC03g03110D* | *AT5G07470* | up | 1.30 | peptidemethionine sulfoxide reductase 3 |
|  | 17 | *BnaA03g02070D* | *AT5G07470* | up | 1.30 | peptidemethionine sulfoxide reductase 3 |
|  | 18 | *BnaC03g03100D* | *AT5G07470* | up | 1.30 | peptidemethionine sulfoxide reductase 3 |
|  | 19 | *BnaA06g12030D* | *AT1G17810* | up | 1.25 | beta-tonoplast intrinsic protein |
|  | 20 | *BnaC05g13770D* | *AT1G17810* | up | 1.22 | beta-tonoplast intrinsic protein |
| C3_43 | 1 | *BnaC08g12450D* | *AT4G27160* | up | 5.68 | seed storage albumin 3 |
|  | 2 | *BnaA08g14120D* | *AT4G27160* | up | 5.03 | seed storage albumin 3 |
|  | 3 | *BnaC05g41990D* | *AT3G11180* | up | 4.97 | 2-oxoglutarate (2OG) and Fe(II)-dependent oxygenase superfamily protein |
|  | 4 | *BnaA05g27810D* | *AT3G11180* | up | 4.96 | 2-oxoglutarate (2OG) and Fe(II)-dependent oxygenase superfamily protein |
|  | 5 | *BnaC04g47140D* | *AT2G03750* | down | -4.90 | P-loop containing nucleoside triphosphate hydrolases superfamily protein |
|  | 6 | *BnaC09g45170D* | *AT5G11420* | down | -4.83 | Protein of unknown function, DUF642 |
|  | 7 | *BnaA06g29670D* | *AT5G49190* | up | 4.76 | sucrose synthase 2 |
|  | 8 | *BnaC07g27090D* | *AT5G49190* | up | 4.72 | sucrose synthase 2 |
|  | 9 | *BnaA07g26590D* | *AT1G67690* | down | -4.54 | Zincin-like metalloproteases family protein |
|  | 10 | *BnaA03g48490D* | *AT4G27160* | up | 4.37 | seed storage albumin 3 |
|  | 11 | *BnaC07g40770D* | *AT4G27160* | up | 4.30 | seed storage albumin 3 |
|  | 12 | *BnaC06g26610D* | *AT1G67690* | down | -4.30 | Zincin-like metalloproteases family protein |
|  | 13 | *BnaA07g24910D* | *AT1G67690* | down | -4.22 | Zincin-like metalloproteases family protein |
|  | 14 | *BnaC06g06810D* | *AT1G54020* | up | 4.06 | GDSL-like Lipase/Acylhydrolase superfamily protein |
|  | 15 | *BnaA06g01410D* | *AT1G54020* | up | 4.01 | GDSL-like Lipase/Acylhydrolase superfamily protein |
|  | 16 | *BnaA03g48460D* | *AT4G27160* | up | 4.00 | seed storage albumin 3 |
|  | 17 | *BnaC06g14680D* | *ATCG00020* | up | 3.94 | photosystem II reaction center protein A |
|  | 18 | *BnaC06g14690D* | *ATCG00020* | up | 3.91 | photosystem II reaction center protein A |
|  | 19 | *BnaA03g35730D* | *AT3G20370* | up | 3.88 | TRAF-like family protein |
|  | 20 | *BnaC06g28770D* | *AT1G67690* | down | -3.87 | Zincin-like metalloproteases family protein |
| C1_51 | 1 | *BnaC06g14680D* | *ATCG00020* | up | 3.54 | photosystem II reaction center protein A |
|  | 2 | *BnaC06g14690D* | *ATCG00020* | up | 3.36 | photosystem II reaction center protein A |
|  | 3 | *BnaCnng12640D* | *ATMG00030* | up | 3.09 | - |
|  | 4 | *BnaC05g37980D* | *ATCG00490* | up | 2.99 | ribulose-bisphosphate carboxylases |
|  | 5 | *BnaC01g27980D* | *ATCG00490* | up | 2.99 | ribulose-bisphosphate carboxylases |
|  | 6 | *BnaC09g27610D* | *ATCG00490* | up | 2.99 | ribulose-bisphosphate carboxylases |
|  | 7 | *BnaA10g13950D* | *ATCG00490* | up | 2.93 | ribulose-bisphosphate carboxylases |
|  | 8 | *BnaA01g34300D* | *ATCG00490* | up | 2.93 | ribulose-bisphosphate carboxylases |
|  | 9 | *BnaC05g37970D* | *ATCG00490* | up | 2.86 | ribulose-bisphosphate carboxylases |
|  | 10 | *BnaC01g27970D* | *ATCG00490* | up | 2.86 | ribulose-bisphosphate carboxylases |
|  | 11 | *BnaC09g27600D* | *ATCG00490* | up | 2.86 | ribulose-bisphosphate carboxylases |
|  | 12 | *BnaC09g26880D* | *-* | up | 2.84 | - |
|  | 13 | *BnaC09g29120D* | *-* | up | 2.84 | - |
|  | 14 | *BnaUnng00820D* | *-* | up | 2.84 | - |
|  | 15 | *BnaC09g16480D* | *-* | up | 2.84 | - |
|  | 16 | *BnaCnng13060D* | *ATCG00065* | up | 2.83 | ribosomal protein S12A |
|  | 17 | *BnaC09g16550D* | *ATCG00065* | up | 2.83 | ribosomal protein S12A |
|  | 18 | *BnaA04g06350D* | *ATCG00065* | up | 2.83 | ribosomal protein S12A |
|  | 19 | *BnaUnng00870D* | *ATCG00065* | up | 2.83 | ribosomal protein S12A |
|  | 20 | *BnaC09g26910D* | *ATCG00065* | up | 2.82 | ribosomal protein S12A |
| C2_52 | 1 | *BnaC05g32150D* | *ATCG00350* | down | -3.48 | Photosystem I, PsaA/PsaB protein |
|  | 2 | *BnaC09g27540D* | *ATCG00350* | down | -3.48 | Photosystem I, PsaA/PsaB protein |
|  | 3 | *BnaC04g15570D* | *ATCG00350* | down | -3.48 | Photosystem I, PsaA/PsaB protein |
|  | 4 | *BnaA05g00900D* | *ATCG00350* | down | -3.48 | Photosystem I, PsaA/PsaB protein |
|  | 5 | *BnaUnng02400D* | *ATCG00350* | down | -3.46 | Photosystem I, PsaA/PsaB protein |
|  | 6 | *BnaC04g15560D* | *ATCG00340* | down | -3.16 | Photosystem I, PsaA/PsaB protein |
|  | 7 | *BnaC09g27530D* | *ATCG00340* | down | -3.16 | Photosystem I, PsaA/PsaB protein |
|  | 8 | *BnaC05g32140D* | *ATCG00340* | down | -3.16 | Photosystem I, PsaA/PsaB protein |
|  | 9 | *BnaC03g60960D* | *ATCG00280* | down | -2.92 | photosystem II reaction center protein C |
|  | 10 | *BnaC09g27520D* | *ATCG00280* | down | -2.92 | photosystem II reaction center protein C |
|  | 11 | *BnaC05g32120D* | *ATCG00280* | down | -2.92 | photosystem II reaction center protein C |
|  | 12 | *BnaC05g32130D* | *ATCG00280* | down | -2.92 | photosystem II reaction center protein C |
|  | 13 | *BnaA10g13950D* | *ATCG00490* | down | -2.84 | ribulose-bisphosphate carboxylases |
|  | 14 | *BnaA01g34300D* | *ATCG00490* | down | -2.84 | ribulose-bisphosphate carboxylases |
|  | 15 | *BnaC05g37980D* | *ATCG00490* | down | -2.77 | ribulose-bisphosphate carboxylases |
|  | 16 | *BnaC01g27980D* | *ATCG00490* | down | -2.77 | ribulose-bisphosphate carboxylases |
|  | 17 | *BnaC09g27610D* | *ATCG00490* | down | -2.77 | ribulose-bisphosphate carboxylases |
|  | 18 | *BnaC09g16500D* | *-* | down | -2.74 | - |
|  | 19 | *BnaA04g18980D* | *AT2G32690* | up | 2.59 | glycine-rich protein 23 |
|  | 20 | *BnaCnng12640D* | *ATMG00030* | down | -2.58 | - |
| C3_53 | 1 | *BnaCnng12680D* | *ATMG00830* | up | 7.13 | cytochrome C biogenesis 382 |
|  | 2 | *BnaC01g26940D* | *ATMG00650* | up | 6.86 | NADH dehydrogenase subunit 4L |
|  | 3 | *BnaCnng12880D* | *ATMG00650* | up | 6.86 | NADH dehydrogenase subunit 4L |
|  | 4 | *BnaC01g27100D* | *-* | up | 6.56 | - |
|  | 5 | *BnaCnng12730D* | *-* | up | 6.56 | - |
|  | 6 | *BnaCnng12720D* | *AT2G07687* | up | 6.48 | Cytochrome c oxidase, subunit III |
|  | 7 | *BnaC01g27090D* | *ATMG00730* | up | 6.45 | cytochrome c oxidase subunit 3 |
|  | 8 | *BnaC04g21290D* | *ATMG01360* | up | 6.38 | cytochrome oxidase |
|  | 9 | *BnaCnng12810D* | *ATMG00665* | up | 6.28 | NADH dehydrogenase 5B |
|  | 10 | *BnaCnng12800D* | *ATMG00513* | up | 6.23 | NADH dehydrogenase 5A |
|  | 11 | *BnaC01g26930D* | *ATMG00640* | up | 6.22 | hydrogen ion transporting ATP synthases, rotational mechanism; zinc ion binding |
|  | 12 | *BnaCnng12890D* | *ATMG00640* | up | 6.20 | hydrogen ion transporting ATP synthases, rotational mechanism; zinc ion binding |
|  | 13 | *BnaCnng13090D* | *ATMG00510* | up | 6.18 | NADH dehydrogenase subunit 7 |
|  | 14 | *BnaCnng12500D* | *ATMG00285* | up | 6.16 | NADH dehydrogenase 2A |
|  | 15 | *BnaCnng13130D* | *ATMG00900* | up | 6.15 | cytochrome C biogenesis 256 |
|  | 16 | *BnaCnng48530D* | *ATMG00510* | up | 6.14 | NADH dehydrogenase subunit 7 |
|  | 17 | *BnaC03g70080D* | *ATCG00490* | up | 6.09 | ribulose-bisphosphate carboxylases |
|  | 18 | *BnaCnng12480D* | *ATMG01360* | up | 6.08 | cytochrome oxidase |
|  | 19 | *BnaCnng12820D* | *ATMG00520* | up | 6.02 | Intron maturase, type II family protein |
|  | 20 | *BnaUnng03950D* | *ATMG01170* | up | 6.01 | ATPase, F0 complex, subunit A protein |
